# Documenting and analyzing pre-reflective self-consciousness underlying ongoing performance optimization in elite athletes: the theoretical and methodological approach of the course-of-experience framework

**DOI:** 10.3389/fpsyg.2024.1382892

**Published:** 2024-06-25

**Authors:** Eric Terrien, Benoît Huet, Paul Iachkine, Jacques Saury

**Affiliations:** ^1^Institut des Sciences du Sport, Faculté des Sciences Sociales et Politiques, Université de Lausanne, Lausanne, Switzerland; ^2^Nantes Université, Movement - Interactions – Performance, MIP, Nantes, France; ^3^Ecole Nationale de Voile et des Sports Nautiques, Beg Rohu, Saint Pierre-Quiberon, France

**Keywords:** empirical micro-phenomenology, enaction, expertise, skilled performance, Athlete’s experience, attentional foci, continuous improvement, windsurfing

## Abstract

Traditional theories of motor learning emphasize the automaticity of skillful actions. However, recent research has emphasized the role of pre-reflective self-consciousness accompanying skillful action execution. In the present paper, we present the course-of-experience framework as a means of studying elite athletes’ pre-reflective self-consciousness in the unfolding activity of performance optimization. We carried out a synthetic presentation of the ontological and epistemological foundation of this framework. Then we illustrated the methodology by an in-depth analysis of two elite windsurfers’ courses of experience. The analysis of global and local characteristics of the riders’ courses of experience reveal (a) the meaningful activities accompanying the experience of ongoing performance optimization; (b) the multidimensionality of attentional foci and the normativity of performance self-assessment; and (c) a micro-scale phenomenological description of continuous improvement. These results highlight the fruitfulness of the course-of-experience framework to describe the experience of being absorbed in an activity of performance optimization.

## 1 Introduction

The optimization of motor skills is crucial in many professional fields, for example to improve productivity or prevent musculoskeletal disorders. In sports, optimizing motor skills is an inherent aspect of the profession for elite level athletes. However, working on the conscious dimensions of the athletes’ movements is often considered at odds with high performance (e.g., Hauw, 2009). Indeed, it is commonly assumed that elite athletes have fully automatized their motor skills and therefore implement them without conscious thinking. In recent years, however, this assumption has been challenged by multiple publications relying on empirical evidence and phenomenological description (e.g., Toner and Moran, 2014; Christensen et al., 2016; Montero, 2016; Toner et al., 2021). These works reject the idea of expert performance being driven by the principle of automaticity, i.e., involving neither self-reflective thinking, nor planning, nor predicting, nor deliberation, nor mental effort (Montero, 2013). In contrast, these works rehabilitate the role of the athlete’s pre-reflective self-consciousness while training to improve performance. This position generates theoretical and methodological challenges to empirically document and analyze athletes’ pre-reflective self-consciousness in the flow of their training or competition activities.

The aim of this paper is (a) to present the theoretical assumptions and methodological procedures that form the main components of a scientific empirical qualitative approach to human activity that gives a central place to the analysis of pre-reflective self-consciousness (i.e., the *course-of-experience* framework, Theureau, 2006; Poizat et al., 2023a), and (b) to present an operationalization of the methodology and share some empirical insights derived from a case study involving elite sailors.

### 1.1 The recent rehabilitation of the importance of mindful processes in the performance and skill optimization of expert athletes

Traditional theories of motor learning emphasize the automaticity of skillful action. Montero (2013) refers to this view as the principle of automaticity. According to this view, expert performance when all is going well involves neither self-reflective thinking, nor planning, nor predicting, nor deliberation, nor mental effort (Montero, 2013). Indeed, Fitts and Posner’s (1967) three-stage model of skill acquisition has been an influential model for scientists and coaches for many decades. According to this model, skill acquisition follows three sequential stages: the cognitive stage, characterized by inaccurate, uncoordinated movements and the use of explicit knowledge; the associative stage, characterized by a more established level of performance and an attention focused on specific details of the sequence of actions required to perform; and the autonomous stage characterized by consistent performance associated with little or no conscious attention, that is, an automatic execution of the skill. Associated to this view is the belief that paying attention to the movement is counterproductive for high-level skill execution (Beilock et al., 2002). Masters’ theory of reinvestment (Masters, 1992; Masters and Maxwell, 2008) suggests that automated motor processes can be disrupted by reinvestment. This concept refers to the manipulation of conscious, explicit, rule-based knowledge, by the working memory to control the mechanics of one’s movements during its execution (Masters and Maxwell, 2004). According to this theory, the disruption of an automated motor process causes a “dechunking” of the movement into a sequence of independent units similar to what it was early on in learning, slowing performance and creating opportunities for error at each transition (Masters, 1992).

However, Toner et al. (2015a) challenged the idea that automaticity is virtuous for skilled performance. The authors highlight that the mindlessness associated with the classical conceptualization of automaticity can be deleterious for skilled performance. They argue that excessive automaticity reduces the athletes’ ability to respond flexibly to performance demands and may lead to mistakes, slips and lapses in skilled performance. Moreover, they highlight that skillful athletes’ practice during training sessions and competitions never amounts to the mere execution of previously automated skills, but also fundamentally consists in a continuous effort to improve these skills, and/or to build new skills in order to optimize performance (Toner and Moran, 2014; Toner et al., 2021). Indeed, the stance adopted by Toner et al. (2021) is that an expert’s ability to execute complex skills under demanding conditions requires embodied skills, conscious awareness of bodily movements and reflective practice. This position is very close to those of diverse authors in the cognitive science domain of skilled action (e.g., Sutton et al., 2011; Christensen et al., 2016; Fridland, 2017; Pacherie and Mylopoulos, 2021; Ivy, 2022). Indeed, Sutton et al. (2011) underlined that in expert skilled performance, patterns of behavior which might appear automated are in fact continually adaptable to the circumstances, emotional states, past meaning, and evolving goals. Pacherie and Mylopoulos (2021) emphasized the role of the representation of actions making possible flexible and efficient skilled action. While the philosophical debate on skilled action remains open, the position in the present paper is that the achievement of high performance is not mindless but is accompanied by an experience lived by the athlete as their own experience. Furthermore, we postulate that this experience can be documented and analyzed through the study of the athlete’s pre-reflective self-consciousness, containing the athlete’s own intentions, expectations, focus of attention, perceptions, domain-specific knowledge mobilization and/or emergence, that accompany their ongoing action at every moment.

### 1.2 The challenges of empirically studying expert athletes’ pre-reflective self-consciousness during their ongoing activity

Researchers facing the challenge of studying an expert athlete’s pre-reflective self-consciousness during their ongoing activity rely on ontological and epistemological presuppositions to answer two fundamental questions: (a) what is the nature of the phenomenon of pre-reflective self-consciousness in ongoing human activity; and (b) how to empirically describe and analyze pre-reflective self-consciousness in “real life” sports situations. In the domain of the qualitative analysis of sports situations, these questions are mostly addressed by referring to a phenomenological approach of cognition and human activity (e.g., Toner et al., 2021; Ravn, 2023).

From an ontological viewpoint, two modes of consciousness (or awareness)^1^ during performance are distinguished by the authors: the pre-reflective and the reflective modes (Toner and Moran, 2014; Toner et al., 2016). The distinction between these two modes can be explained as follows: the pre-reflective mode is when the experience is lived through directly, without being objectified or distanced through reflective thought. Colombetti defines pre-reflective self-awareness as “one’s self is experienced or lived through as the subject of awareness, without any process of reflection on itself.” (Colombetti, 2011, p. 303). Conversely, reflective self-awareness, is when “one’s self is reflected upon and thus *objectified*—as, e.g., when one considers his or her own intentions or actions to assess whether they are appropriate to a certain situation.” (Colombetti, 2011, pp. 302–303). Furthermore, Legrand (2007) defines pre-reflective self-consciousness emphasizing that it is a constant feature of conscious experience: “it is a constant structural feature of conscious experience, and corresponds to the consciousness of the self-as-subject that is not taken as an intentional object” (Legrand, 2007, p. 584). That is, pre-reflective and reflective consciousness do not mutually exclude each other. Instead, according to Sartre’s conception of pre-reflective self-consciousness, it is the pre-reflective consciousness which renders the reflection possible (Sartre, 2003; Zahavi, 2008). In the present paper, we rely Sartre’s conception of pre-reflective self-consciousness s an ongoing, immediate understanding of the lived experience (*le vécu*) that silently accompanies it (Sartre, 2003; Theureau, 2006).

Therefore, from an epistemological viewpoint, the main challenges that emerge through the empirical investigation of expert athletes’ pre-reflective self-consciousness during their ongoing activity are twofold. Firstly, the investigation presupposes adequate methods to gain access to the perspective of the actors whose ongoing situated activity is being studied. For researchers, accessing to the actors’ perspective is made possible by a dazzling multiplicity of methods (Høffding, 2023). Several overviews of these methods have been previously published (e.g., Høffding et al., 2023; Lumma and Weger, 2023). In sports, common methods include thinking aloud (e.g., Eccles and Arsal, 2017), event-focused interviews (e.g., Jackman et al., 2022), autoethnography (e.g., Allen-Collinson and Hockey, 2008), explicitation interviews (e.g., Mouchet et al., 2019) and self-confrontation interviews (e.g., Hauw and Durand, 2007). All these methods contribute to the documenting of athletes’ experiences, not all of them are suitable to focus on the content of the athlete’s pre-reflective self-consciousness during the unfolding of the action. Firstly, this documentation requires methods that help the athlete to “re-live” the past situation while avoiding the athlete’s expression of reflective consciousness during the interview (Poizat et al., 2023b). The self-confrontation interview method presented in this article aims to create favorable conditions for the expression of pre-reflective consciousness. It aims to the explicitation of initially implicit layers of experience that go deeper than the descriptions typically made by athletes in their practice but that also tend to be expressed spontaneously during the interviews. By confronting the actor with concrete and “objective” traces of their activity and resorting to questioning aimed at describing their experience, the self-confrontation interview promotes precise mnemonic recall of past experiences while allowing a control of retrospective reconstructions or rationalizations by the actor to limit them. Secondly, investigating athletes’ pre-reflective self-consciousness requires a set of articulated notions or concepts to analyze the data collected and to produce empirical evidence about this activity.

The theoretical and methodological approach of the course-of-experience is designed to address these ontological and epistemological challenges, in order to specifically study the dynamics of the pre-reflective self-consciousness accompanying human activity (i.e., the “course-of-experience”), of which sport situations can be part.

## 2 The course-of-experience theoretical and methodological framework

The course-of-experience framework was initially developed in France in the area of work analysis in cognitive ergonomics in the 1980s (Theureau, 2003; Poizat and San Martin, 2020; Poizat et al., 2023a). Over the past two decades, it has undergone successive theoretical refinements concomitantly to its extension to a diversity of research domains. To date, dozens of published studies have referred to this framework in the area of sports science (Hauw, 2009; Bourbousson et al., 2010; Poizat et al., 2012; Sève et al., 2013; R’Kiouak et al., 2016, 2018; Seifert et al., 2016, 2017; Rochat et al., 2017; Terrien et al., 2020, 2022). However, the main publications of Theureau on the theoretical development of the course-of-experience framework have been published in French-language handbooks (Theureau, 1992, 2004, 2006, 2009, 2015). Recently an effort has been made to internationalize this framework by publishing updated syntheses of its progress and contributions in English (Poizat and San Martin, 2020; Poizat et al., 2023a). In the present paper, we provide an in-depth description of this research program’s concrete methodological implementation and its empirical interest in studying the pre-reflective dimensions of the activity of elite athletes.

### 2.1 Course-of-experience as a theoretical object to empirically analyze pre-reflective self-consciousness

Consistent with its ontological and epistemological assumptions,^2^ this theoretical framework aims to enable an empirical analysis of human activity in a way that gives primacy to the first-person experience of the actors. From this perspective, studying human activity requires to document the subject’s continuous sense making activity that arises through their interaction with their environment and giving rise to their own world, or in other terms, the history of pre-reflective self-consciousness, what constitutes the theoretical object of the course-of-experience (Theureau, 2006; Poizat et al., 2023a).

As for the methodological implications, Theureau (2006) postulates that the course-of-experience can be studied empirically to the extent that under favorable conditions an actor can show (by miming or gesturing), relate and comment on the content of their pre-reflective consciousness to an observer-interlocutor. Favorable conditions include a set of ethical and contractual conditions, and the use of methods such as self-confrontation interviews (Theureau, 2003, 2006). These conditions and methods are illustrated in the case study presented in this paper.

### 2.2 General features of the methods aiming to analyze the course-of-experience

Besides the fundamental hypothesis of the course-of-experience as the history of pre-reflective self-consciousness (Theureau, 2006; Poizat et al., 2023a), the course-of-experience framework provides researchers with a set of methods for data collection, and a generic analytical model of the components of the course-of-experience, to guide the data processing.

The methods of data collection aim, on the one hand, to provide researchers with a fine understanding of the spatio-temporal, social and cultural context of the actors’ activity, and on the other hand, to create favorable conditions for the expression of verbalizations referring to the actor’s pre-reflective self-consciousness.

These methods include typically three types of data collection (Poizat et al., 2023a): (a) field notes, ethnographic observations and preliminary interviews to familiarize the researchers with the situation and with the practice under study; (b) *in situ* continuous video recordings during the situation to be analyzed, to provide precise behavioral and contextual information about the ongoing activity; and (c) self-confrontation interviews, based on the *in situ* video recordings, aiming to have the participant “re-live” the situation and express as naturally as possible what they aimed for, did, expected, felt, thought, and perceived during the past experience and practice, in order to document their pre-reflective self-consciousness during this practice. It should be noted that partial expression of pre-reflective self-consciousness can sometimes occur during the unfolding of the activity through verbal communication or spontaneous thinking aloud. Therefore, when the video recordings are made using on-board cameras capturing the verbalizations of the actors, these also contribute to documenting the content of the actors’ pre-reflective self-consciousness during their practice. It is noteworthy that prior to the implementation of these methods, the researchers and participants must agree on the study’s ethical and contractual conditions, and on the practical conditions of its concrete implementation, taking into account the specific constraints of the situations to be analyzed.

The generic model of analysis of the course-of-experience framework, guiding the data processing, is based on the hypothesis that lived experience can be broken down from a continuous stream into discrete units assumed to be the expression of signs. This model of analysis is inspired by Peirce’s “thought-sign” hypothesis and made consistent with enactive assumptions to analyze human activity as an “activity-sign” (Poizat and San Martin, 2020; Poizat et al., 2023a; see also Poizat et al., 2023b for a discussion about the use of Peircean semiotics in this framework). The generic model of the *hexadic sign* offers a coherent system of descriptive components that allow a fine-grained empirical documentation of the course-of-experience, i.e., the history of pre-reflective self-consciousness. In other words, the six categories of the hexadic sign constitute a pre-established interpretation matrix relating to the different components of an actor’s activity, considered from the actor’s point of view (see the section “Data analysis” below).

In the following section, we rely on a case study drawn from ongoing empirical research to present and illustrate the main steps of this method.

## 3 Methods to document and analyze athletes’ course-of-experience: a case study

These methods are presented and systematically illustrated through a case study drawn from a larger project aiming to identify elite sailors’ typical experiences in relation to the mechanical characteristics of their equipment to improve their speed performances.

### 3.1 Studying elite windsurfers during speed-tests

**Figure 1:**
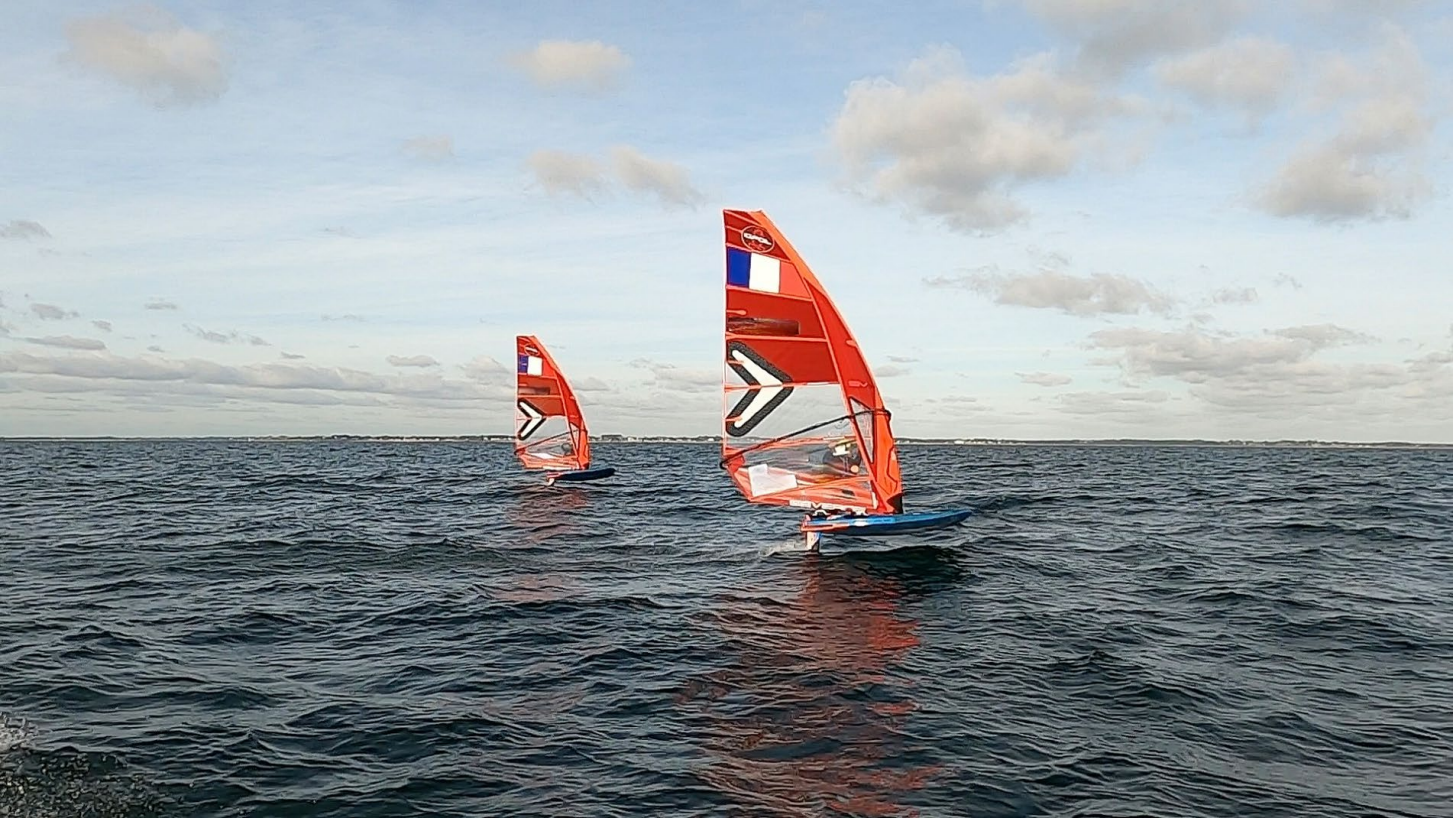
Picture of two IQfoil riders during a speed test. This picture illustrates the wind, sea and weather conditions during the data collection. We can see that both riders are “flying” above the sea surface, supported by their foil.

The case study involved two IQfoil^3^ windsurfing elite male riders performing a speed test in a training session (Figure 1). The latter took place as part of the preparation of French elite riders for the 2024 Paris Olympics. Speed-tests are a typical training situation during which riders sail next to each other for about 2 min to compare their speed, with the goal of sailing as fast as possible. In this situation, riders are *a priori* focused on technical aspects of performance (i.e., motor control, equipment settings), more than on tactical or strategical aspects of performance. The IQFoil windsurfing class is equipped with a hydrofoil allowing it to fly above the surface of the water, and men use a 9m^2^ sail. Using IQfoil equipment requires fine technical skills. Riders must continuously regulate the complex interaction between forces generated by the sail and the hydrofoil. To do so they continuously use their whole body to change the sail position, apply or relieve weight on the board, to regulate their speed while keeping the board above the water surface.

At the time of the data collection, the two participating riders—identified in this article by the pseudonyms of Luca and Adam—were 27 years old and 31 years old, respectively. Both were “successful-elite” athletes, according to Swann et al.’s classification (Swann et al., 2015) and belonged to the French national IQfoil team.

### 3.2 Ethical and contractual conditions of the athlete-researcher collaboration

The study’s ethical conditions were approved by the Ethical Committee for Non-Interventional Research of the authors’ university (approval number 20102020). Both riders provided written informed consent to participate in the study. In addition to these legal precautions applicable to any research, specific ethical and contractual conditions of the athlete-researcher collaboration have been defined for this study, considering the aims of the broader research project of which it was a part. This project was designed in collaboration with the French national sailing team. It aimed, on the one hand, at producing scientific knowledge of the perceptual experiences accompanying the athletes’ interactions with their sport equipment in situations of performance optimization. It also intended to design performance training and optimization aids for French athletes in the context of preparing the 2024 Olympic Games. This project was initiated as an extension of a long-term collaboration (over eight Olympiads) bringing together coaches and athletes from the French national sailing team, researchers, engineers and data-scientists. This collaborative history has helped to build up relations of both mutual trust and mutual knowledge between these protagonists, allowing a fluid and fruitful collaboration in the present study. For instance, the practical conditions for collecting data during training sessions for the present study were the subject of prior discussions between athletes, coaches and researchers in order to best consider the constraints of real-life training situations. These discussions resulted in full adherence and mutual agreement between the protagonists, in relation to these methodological conditions.

### 3.3 Data collection

#### 3.3.1 Field notes, ethnographic observations and preliminary interviews

A set of sequences of observation of training sessions was carried out prior to data collection. The researchers’ objective was to familiarize themselves with the particularities of IQFoil class, newly selected for the 2024 Olympics. These sequences gave rise to multiple discussions and informal interviews with athletes and coaches. During these interactions, the researchers asked questions about the characteristics of this specific equipment and the technical concerns emerging in relation to the flying mode of sailing. This allowed the researchers to make sure they understand the different possibilities of settings of the IQfoil equipment (i.e., foil, board, sail) as well as the vocabulary associated with these settings. Athletes and coaches pointed out the importance of foil settings in the precision of flight stability control, and the importance of balancing weight on the feet for flight regulation.

#### 3.3.2 Collecting *in situ* data about an athlete’s behavior and situational circumstances of an athlete’s activity

Data were collected during a collective training session of the French IQFoil team. For the purposes of this case study, we will focus on the data relating more specifically to one of the speed-tests that was carried out during this training session, concerning the respective activities of Luca and Adam during this speed-test. The speed-test was upwind on port tack, lasting 2 min 40 s, and it took place during the first part of the training session, following a warm-up sequence and a first speed-test. Wind speed was approximately 12 knots during the test. The wind created small waves, and there was no significant ground swell in the sailing area. The picture in Figure 1 was taken during the data collection. IQfoil elite riders are trained to sail in wind between six and 30 knots. Therefore, weather conditions on the day of data collection presented no particular challenges for the riders.

Luca’s and Adam’s behavior were video-recorded from the coach boat during the entire training session (and *a fortiori* during the speed-test of interest for the present case study). Each rider was equipped with an action sport camera (VIRB XE, Garmin) fitted on their helmet, providing a continuous recording of the situation from a first-person point of view. A microphone integrated into the helmet allowed the riders’ verbalizations to be recorded. The athletes were asked to vocalize their thoughts while sailing as long as it did not interfere with their performance optimization activity. The recordings of these *in situ* comments were of great interest, on the one hand, for how they contributed to documenting the riders’ pre-reflective self-consciousness during their activity (e.g., when the rider said, “Here we go, I’m on my way!.. Good downforce in the harness lines… Rather well-balanced, I’m light on the arms”), and on the other hand, for “tagging” precisely the situational circumstances of the riders’ activities (e.g., “Puff to come!” when a wind gust was expected by the rider).

#### 3.3.3 Self-confrontation interviews

Individual self-confrontation interviews were carried out with each rider 2 to 4 h after the training session. This delay was necessary for the riders to have enough time to return to shore, stow their equipment, put warm clothe, and for the researchers to prepare the set up for the self-confrontation interviews (e.g., setting up the interview room, downloading audio-visual recordings). These interviews consisted in confronting each rider with the recorded videos of the training session and to invite them to “re-live” the situation observed and to describe their past experience moment to moment. These interviews lasted approximately 90 min for each rider, of which about 15 min specifically concerned this case study’s speed-test. These interviews share similarities with explicitation interviews (Vermersch, 1994) and micro-phenomenological interviews (Petitmengin et al., 2019) in its ambition to collect descriptions of the micro-dynamics of experience and the conduct of the questioning combining active guidance and non-directiveness (Poizat et al., 2023b). A key difference is to make systematic use of traces of the past activity (e.g., video-recording) as a support to dynamically re-situate the athlete in the past situation (Poizat et al., 2023b). The researcher and the rider were installed in front of a computer playing in sync the videos recorded from the action sport camera and the coach’s boat. The researcher used prompts to guide the riders in a chronological description of the re-lived experience, so that they expressed as precisely as possible what they had aimed for, done, expected, felt, thought, and perceived at every moment. Typical prompts used by the researcher were: “at this moment, what are you doing?,” “what are you looking to do?,” “what are you thinking?,” “what are you focused on?,” or “what are you feeling?” The participants’ answers could then be the subject of requests for more details in order to obtain the most accurate description possible of their experience. Moreover, like the researcher, athletes were able to control video playback by pausing or replaying sequences, and thus to take the time to describe their “re-lived” experience of these moments.

### 3.4 Data analysis: reconstructing and analyzing the athlete’s course-of-experience

The reconstruction of the courses of experience of Luca and Adam (i.e., the history of their pre-reflective self-consciousness) during the speed-test was carried out following four main steps: (a) preparation of a protocol for the analysis of the course-of-experience, (b) identifying the components of the hexadic signs, (c) identifying significant structures of the course-of-experience, and (d) characterizing the course-of-experience in relation with the specific objects of the empirical study (see Appendices A–C for empirical data and analysis).

#### 3.4.1 Preparation of a protocol for the analysis of the course-of-experience

The preparation of the protocol for the analysis of each rider’s course-of-experience consisted of fully transcribing their recorded *in situ* spontaneous comments and their recorded retrospective comments from the self-confrontation interviews. All the significant events occurring during the training session and the main elements of the context of the riders’ activity were also concisely described. This information and the transcripts were then synchronized in a table in order to obtain a chronicle of each rider’s activity during the training session. The three columns of the table contain respectively: (a) the training session timeline, (b) the *in situ* described events and spontaneous comments, and (c) the corresponding verbalizations during self-confrontation interviews. Appendix A presents excerpts from a protocol for the analysis of Luca’s and Adam’s courses of experience.

#### 3.4.2 Identifying the components of the hexadic signs

As pointed out above, the generic model of analysis of the course-of-experience framework is based on the hypothesis that lived experience can be broken down from a continuous stream into discrete units assumed to be the expression of signs. The generic model of the hexadic sign offers a system of descriptive categories of the components of pre-reflective self-consciousness. The hexadic signs are composed by six components (Theureau, 2006): the *Meaningful Units* of the course-of-experience; the *Involvement in the situation* (i.e.*, actor’s concerns or intentions*), the *Anticipation structure* (i.e.*, actor’s expectations*), the *Referential* (i.e.*, actor’s activation of usual knowledge*),^4^ the *Representamen* (i.e.*, perceptual and proprioceptive cues meaningful for the actor*) and the *Interpretant* (i.e.*, process of actor’s validation or invalidation of usual knowledge, or enacting new elements of generality, or types*). These components are identified and documented through a meticulous and iterative interpretive analysis of the data.

Below, we introduce each component and describe the steps we follow to identify their content. Excerpts of Adam’s and Luca’s hexadic signs are presented, respectively, in Tables 1, 2.

**Table 1:** Excerpt of Adam’s hexadic signs.

Time (hh:m:ss)	Meaningful units (MU)	Involvement	Expectations	Referential	Representamen	Interpretant
14: 52: 48	A_MU3: Accelerates in the gust while countering the sail and telling himself that he has reached the limit of the sail power allowing him to remain efficient	- To obtain and maintain an optimal VMG - To use the gust to accelerate - To evaluate the need (or not) to adjust the setting of the sail.	- Acceleration in the gust - Need (or not) to flatten again the sail (applying more outhaul) - Too much effort (or not) to “counter” the sail power	- To accelerate the sail must not be too powerful - Typical indicators of sail power: back hand traction, leech opening - On a short course, I can withstand the effort required	- Optimal speed - High traction in the back hand (powerful sail) - The sail slightly backwinded but remains “bearable” (allows to withstand and to maintain an optimal speed) - Feeling of imbalance between the tractions in the two hands - Sensations of effort and physical fatigue to “counter” the overpower	- It is not necessary to reduce the power of the sail in these conditions to reach optimum speed, as long as it is possible to “counter” the overpower
14: 53: 20	A_MU4: Visually checks the other riders and then his sail, telling himself that he is in a “good phase” of speed	- To assess his trajectory in relation to the other riders - To visually check the sensation of having a “sail well settled”	- Maintaining a good speed in the gust - Variation of his placement and speed in relation to the other riders during the gust	- Variations in wind strength and/or direction can benefit more or less to the rider depending on their placement	- Good phase in speed: possibility of luffing more than Luca and the other riders - Gain more ground to windward than Luca in the gust	- The advantage taken over the other riders is due to good technical use of the gust (allowing him to luff and gain more ground windward), and not to a wind shift (“alignment”)
14: 53: 38	A_MU5: Releases outhaul tension during the lull, while controlling his position in relation to the other riders	- To adapt his sail settings to the decrease of the wind to maintain speed	- Marked slowdown in the lull - Loss of flight height	- The foil loses power in the lull (tendency to “go down a little”) - Releasing the outhaul tension restores power in the sail in the lulls (when the wind dies down)	- Entering a lull, slowing down, loss of power in the sail - Loss of flight height	- Technical adaptation-type: releasing outhaul restores power in the sail

**Table 2:** Excerpt of Luca’s hexadic signs.

Time (hh:m:ss)	Meaningful units (MU)	Involvement	Expectations	Referential	Representamen	Interpretant
14: 49: 53	L_MU1: Position himself leeward of the other riders, on a same line	- To prepare for the start of the speed test - To be on par with the other riders at the start	- All riders on a same line	- Typical procedure: a speed test start requires that all riders are on a sane line, perpendicular to the wind	- Gathering of all riders, placed on the same line perpendicular to the wind - Is placed leeward of the fleet	- Procedure-type of starting a speed test
14: 51: 48	L_MU2: Pump and start flying while observing the other riders	- To position himself and control the flight before the start of the speed test - To stay on par with the other riders	- All riders flying on a same line, ready to start the speed test	- Starting a speed test in IQF requires all riders flying	- Riders flying - All placed on a line perpendicular to the wind	- Procedure-type of starting a speed test
14: 52: 10	L_MU3: Starts the speed test - « *Ok let’s go* »	- To obtain and maintain an optimal VMG - To take the start of the speed test	- Optimal VMG	- The speed test begins when all riders are flying on a same line	- All riders flying - All riders get going	- Procedure-type of starting a speed test
14: 52: 17	L_MU4: Assess his speed in relation to the others (look at the others “*Pretty slow so far*”)	- to estimates his performance (speed, windward ground gain) compared to the windward riders	- Position of advantage over the other riders	- The windward ground gained is estimated in relation to a perpendicular to the wind - All riders may not benefit from the same wind during a speed test	- Slower than the Other riders - Light wind	- Slower speed that the other riders - Wind “lighter” for him than for the others
	L_MU5: Negotiates the entry into a puff by adapting his outhaul setting	- To make good use of the puff to optimize his VMG - To adjust the sail settings to higher wind conditions	- Incoming puff - Neutral sail, settled, allowing to be well balanced and to accelerate when entering the puff	- Important to adapt the settings to the wind variations - A well set sail = neutral and centered in the harness lines, allowing easy sheet in/sheet out, and good transmission of the thrust to the board	- Well balanced sail, stable, “solid” - Board acceleration	- Optimal sail settings indicators-types

The first step is to identify *meaningful units* (MU) of the course-of-experience. The MU is a segmentation into discrete units (e.g., a practical action, a communication, a thought) of the continuous stream of the actors’ actions. In other terms, the chain of MUs offers a first view of the “story” that is experienced by the actor in the situation. Adam’s and Luca’s courses of experience during the speed-test was made up of 10 MU and 12 MU, respectively (Appendix B). For example, the fourth MU for Luca (L_MU4) was “Assess his speed in relation to the other riders” (at the same time, he looked at the other riders and spontaneously said *in situ*: “Pretty slow so far”). This occurred just after L_MU3, labeled “Begins the speed-test,” and just before L_MU5, labeled “Negotiates the entry into a puff by adapting his outhaul setting” (Table 2).

The following step is to identify the five other components of each hexadic sign.

The *Involvement in the Situation* describes *a nebula of openings* (Poizat et al., 2023a) that circumscribes a certain range of possibilities in terms of actions. Involvement in the situation is derived at each given moment from the previous history of coupling between the actor and their situation. It can be documented empirically in terms of concerns, or intentions, orienting the actors’ activity in the situation. For example, the involvement in the situation of Luca underlying his activity associated with L_MU4, was “to estimate his performance (speed, windward ground gain) compared to the windward riders” (Table 2).

The *Anticipation Structure* corresponds to the actor’s expected events and actions at a given moment, considering their involvement in the situation. These expectations can be passive (e.g., waiting for events) or active (e.g., anticipating the events according to ongoing action and intentions). For example, Luca’s expectations associated with L_MU4, were: [expectation of] “position of advantage over the other riders” (Table 2).

The *Referential* corresponds to the usual knowledge, which is implicitly or explicitly activated by the actor at a given moment, regarding their involvement in the situation and their anticipation structure. The referential belongs to the actor’s own habits, constructed during past interactions between the actor and their environment. For example, the elements of the referential documented for Luca at the moment corresponding to his L_MU4 was: “the windward ground gained is estimated in relation to a perpendicular to the wind,” and “all riders may not benefit from the same wind during a speed-test” (Table 2).

The *Representamen* corresponds to the elements that are significant to actors at a given moment of their interaction with their environment, considering the structure of preparation formed by the involvement in the situation, the anticipation structure and the referential.^5^ These elements could be perceptive, proprioceptive or mnemonic, and correspond to salient “perturbations” or “shocks” considered by the actor during their activity. For example, L_MU4 was associated with two perceptual meaningful elements: “to be slower than the other riders,” and the feeling of “light wind” (Table 2).

The *Interpretant* refers to the validation, invalidation or emergence of knowledge. This is based on the hypothesis that any human activity is accompanied by a learning process leading to the validation or invalidation of usual knowledge, and/or to the emergence of new knowledge. For example, L_MU4 was associated with the emergence of the knowledge that Luca had a “slower speed than the other riders” at this moment, generating the hypothesis that a “wind lighter for him than for the others” could explain these speed differences (Table 2).

The presentation of riders’ courses of experience in the form of hexadic signs tables provides a readable and systematic description of the riders’ experience during the unfolding of the action. In Tables 1, 2 (see also Appendix B), each line represents a hexadic sign and allows for understanding the riders’ experience at that moment by relating what they did (MU) with their Involvement, Expectations, Referential, Representamen and Interpretant at that same moment. Reading the tables from top to bottom provides a view of the history of the riders’ experience that can be related to the evolution of their Involvement, Expectations, Referential, Representamen and Interpretant.

#### 3.4.3 Identifying significant structures of the course-of-experience

The previous step consisted in describing the local dynamics of the course-of-experience. The present step aims to identify the global structure of the course-of-experience. Indeed, the MUs and their components link together and fit into larger elements of the course-of-experience, the “episodes” (or “sequences”), which themselves form higher-level meaningful stories. Depending on the flow of events experienced by each actor, the length and number of episodes can vary. Their identification results from a qualitative analysis by the researcher of the temporal coherence of the actors’ involvements in the situation, revealing global concerns. Each episode starts with a global concern of the actor, and ends when the expectations linked to this concern are satisfied, or when another concern is opened in response to a particular event. For example, in Adam’s course-of-experience, the transition between the episode “Controls the flight to be on the same line as the other riders to start the speed-test” (A_Ep1), and the episode “Experiences a good phase while reaching the limit of the sail power during a gust” (A_Ep2) occurs when Adam’s involvement and expectations are no longer related to the start of the speed-test, but are related to the optimization of his performance in a gust. Table 3 presents an illustration of the process of identifying episodes from Adam’s course-of-experience, and Table 4 presents an overview of all the episodes identified in Adam’s and Luca’s courses-of-experience.

**Table 3:** Illustration of the process of identifying episodes from Adam’s course-of-experience.

Episode	Meaningful units (MU)	Involvement	Expectations
A_Ep1 Controls the flight to be on the same line as the other riders to start the speed test	A_MU1	- To take the start of the speed test - To control the flight before the start of the speed test - To stay on the same line as the others for the start	- All riders flying on a same line, ready to start the speed test (“is it good?”)
A_EP2 Experiences a good phase while reaching the limit of the sail power during a gust	A_MU2	- To reduce the “power” of his sail before hitting the gust	- Is going to enter in gust - The gust will “overpower” his sail
	A_MU3	- To obtain and maintain an optimal VMG - To use the gust to accelerate - To evaluate the need (or not) to adjust the setting of the sail.	- Acceleration in the gust - Need (or not) to flatten again the sail (applying more outhaul) - Too much effort (or not) to “counter” the sail power
	A_MU4	- To assess his trajectory in relation to the other riders - To visually check the sensation of having a “sail well settled”	- Maintaining a good speed in the gust - Variation of his placement and speed in relation to the other riders during the gust
A_Ep3 Reacts to a loss of speed in a lull by releasing outhaul	A_MU5	- To adapt his sail settings to the decrease of the wind to maintain speed	- Marked slowdown in the lull - Loss of flight height

**Table 4:** Overview of Adam’s and Luca’s episodes of meaningful activity during the speed test.

Adam		Time of the day	Luca	
Episodes	MUs		Episodes	MUs
A_Ep1: Controls the flight to be on the same line as the other riders to start the speed test	A_MU1	14:52:00	L_Ep1: Gets up to speed and controls the flight to be on the same line as the other riders to start the speed test	L_MU1 L_MU2
A_Ep2: Experiences a good phase while reaching the limit of the sail power during a gust	A_MU2 A_MU3 A_MU4		L_Ep2: Reaches an optimal state of operation in a gust	L_MU3 L_MU4 L_MU5 L_MU6
		14:53:00	L_Ep3: Adjusts the sailing mode to a wind shift	L_MU7 L_MU8
A_ Ep3: Reacts to a loss of speed in a lull by releasing outhaul	A_MU5		L_Ep4: Reacts to a lull by releasing outhaul to regain power	L_MU9
A_Ep4: Reacts to an unexpected stall of the sail	A_MU6 A_MU7	14:54:00	L_Ep5: Experience of difficulties to maintain the flight in the lull	L_MU10
A_Ep5: Setting the sail to make good use of a puff	A_MU8		L_Ep6: Makes good use of a puff to accelerate	L_MU11
A_Ep6: Makes a global assessment of the speed test	A_MU9	14:55:00	L_Ep7: Draws conclusions about the speed test	L_MU12

The timeline is positioned for illustrative purposes to represent the temporal evolution of the riders’ courses of experience. For precise information on the time markers, refer to Appendix B.

## 4 Empirical characterization of pre-reflective self-consciousness underlying athletes’ ongoing performance optimization

As presented in the previous section, the general ambition of the theoretical and methodological framework of the course-of-experience is to provide a set of analytical notions and methods that make it possible to finely describe the experience of actors engaged in their practice. As such, the analyses of the course-of-experience are likely to shed light on multiple topics and themes of interest for researchers. Indeed, previous works carried out within this theoretical and methodological framework in the sports field have empirically characterized phenomena such as interpersonal coordination processes within team sports teams or crews (e.g., Bourbousson et al., 2010; Poizat et al., 2012; R’Kiouak et al., 2016), the dynamics of knowledge sharing between teammates (e.g., Bourbousson et al., 2011), the improvised adaptation of athletes to the contingencies of competitions (e.g., Hauw and Durand, 2007; Hauw et al., 2008), the implementation of reflective practice by athletes in training and in competition (e.g., Hauw, 2009), the construction of knowledge during competitions (e.g., Sève et al., 2002), or the interactions between athletes and their sports equipment in the search for performance (e.g., Terrien et al., 2022).

The purpose of the present section is to show what kind of insights can be gained by analyzing athletes’ courses of experience, regarding their activity of ongoing performance optimization. Three characteristics of this activity are presented and briefly discussed in the following sub-sections: (a) meaningful activities accompanying the experience of ongoing performance optimization; (b) multidimensionality of attentional foci and the normativity of performance self-assessment; and (c) a micro-scale phenomenological description of continuous improvement. These themes are chosen because of the topicality of the debates they are subject to in the current context of research in sports psychology, particularly regarding the cognition of the athletes in their pursuit of performance.

### 4.1 Meaningful activities accompanying the experience of ongoing performance optimization

Within the course-of-experience framework, meaningful activities experienced by an athlete engaged in a sport situation can be revealed by a global analysis of the episodes structuring the athlete’s course-of-experience (e.g., Sève et al., 2002; Rochat et al., 2017; Terrien et al., 2020, 2022). In the present study, we identified six episodes in Adam’s course-of-experience and seven episodes in Luca’s course-of-experience (Table 4). Then, we categorized Luca’s and Adam’s episodes into three main categories, representing three types of meaningful activities accompanying the riders’ experience of ongoing performance optimization during the speed-tests studied (Table 5).

**Table 5:** Categorization of types of episodes structuring the riders’ activity of performance optimization during the speed test.

Types of episodes	Episodes
Reach and maintain optimal performance	A_Ep1: Controls the flight to be on the same line as the other riders to start the speed test A_Ep2: Experiences a good phase while reaching the limit of the sail power during a gust A_Ep5: Sets the sail to make good use of a puff L_Ep1: Gets up to speed and controls the flight to be on the same line as the other riders to start the speed test L_Ep2: Reaches an optimal state of operation in a gust L_Ep6: Makes good use of a puff to accelerate
React to perturbation and recover	A_Ep3: Reacts to a loss of speed in a lull by releasing outhaul A_Ep4: Reacts to an unexpected stall of the sail L_Ep3: Adjusts the sailing mode to a wind shift L_Ep4: Reacts to a lull by releasing outhaul to regain power
Reflectively analyze the speed test situation	A_Ep6: Makes a global assessment of the speed test L_Ep7: Draws conclusions about the speed test

The first category was labeled *“Reach and maintain optimal performance.”* The episodes within this category are characterized by concerns and expectations related to the possibilities of reaching and maintaining “good phases” by making good use of environmental conditions (e.g., a gust).

The second category was labeled *“React to perturbation and recover.”* The episodes within this category are characterized by concerns and expectations related to the perception of a decrement in performance, triggering actions to recover and avoid losing speed.

The third category was labeled *“Reflectively analyze the speed-test situation.”* The episodes within this category are characterized by concerns and expectations related to the realization at a given instant of an assessment of the progress of the situation up to this instant, to build knowledge about it.

As explained above, a global analysis of the riders’ experience identifies a variety of meaningful activities accompanying the experience of ongoing performance optimization. The identification of three types of meaningful activities during the speed-test is in line with previous studies highlighting the variety of activities in which the athletes are engaged when seeking performance (e.g., Sève et al., 2002; Hauw et al., 2003; Terrien et al., 2020). The riders’ capacity to switch between these activities illustrates their adaptability to the performance environment. For example, during the speed test, both riders sailed through a gust followed by a lull that they experienced at the same time. During this gust, Adam experienced a good phase while reaching the limit of the sail power (A_Ep2). As he entered the lull, he had to react to the loss of speed inherent to the decrease in wind strength by releasing the outhaul (A_Ep3), therefore switching from an activity categorized as “Reach and maintain optimal performance” to an activity categorized as “React to perturbation and recover.” Luca’s case is slightly different. While Luca also experienced a good phase during the gust of wind (L_Ep2), he switched to an activity of reacting to a perturbation to negotiate a roll of wind (L_Ep3) approximately 30 s earlier before reacting to the lull similarly to Adam (i.e., releasing outhaul, L_Ep4). Following the lull, both riders switched back to an activity categorized as “reach and maintain optimal performance” (A_Ep5, L_Ep6). The fact that the adjustment to the roll of wind was only made by one rider can be explained by the localized nature of some wind variation, which can affect differently the riders even when separated by only a few meters. Indeed, IQfoil riders are immersed in an uncertain and fluctuating outdoor environment, however, the athlete’s capacity to interact efficiently and flexibly with the situation (i.e., instead of invariantly executing a planned routine and automated technical skills) has previously been pinpointed in more “stable” environments (Hauw et al., 2003, 2008).

Furthermore, the capacity to switch between different levels of cognitive activity (e.g., pre-reflexive, ongoing activity of performance optimization or reaction to perturbation, and reflective analyzes of the situation) may be seen here as a characteristic of expert skilled performance (Sutton et al., 2011; Toner and Moran, 2014; Toner et al., 2015b). For example, just before the end of the speed test, Adam switched from an activity of reaching and maintaining optimal performance to an activity of reflectively analyzing the speed test situation by making a global assessment of the leg, self-analyzing what he had just done, felt and learned (A_Ep6). In the present case study, the duration of the speed test was short (less than 3 min) and this switch occurred for the riders toward the end of the speed test. A previous study in sailing highlighted that switching to reflective mode can occur several times during speed tests of longer duration (Terrien et al., 2020).

### 4.2 Multidimensionality of attentional foci and the normativity of performance self-assessment

Numerous studies in sport science have sought to understand the links between the athletes’ attentional foci and performance (Bernier et al., 2009, 2011, 2016; Wulf, 2013). In most of this research, however, the attentional focus is imposed by experimental conditions and is associated with discrete actions that may not represent a natural performance situation (Bernier et al., 2011, 2016). Within the course-of-experience framework, a local analysis of the athletes’ experiences provides information on the elements of the situation on which the athletes direct their attention. Among the components of the hexadic sign, the anticipation structure (particularly the perceptual expectations) and the representamen are the main components that provide information, respectively about the elements of the situation to which the athlete is particularly sensitive, and about the elements of the situation that capture the athlete’s attention at each moment. To exemplify, in the present study we proceeded to identify the typical contents of the riders’ representamen (Tables 6, 7).

**Table 6:** Categorization of Adams’ typical representamen.

MU	Representamen	Typical contents of the Representamen
A_MU1	- Positioning on the same perpendicular line to the wind in a leeward position of the fleet, and windward of Luca	Maintaining position in the fleet
A_MU2	- Incoming gust	Increasing wind
A_MU3	- Optimal speed - High traction in the back hand (powerful sail) - The sail slightly backwinded but remains “bearable” (allows to withstand and to maintain an optimal speed) - Feeling of imbalance between the tractions in the two hands - Sensations of effort and physical fatigue to “counter” the overpower	Good speed High sail power Perturbating sail movements Transmission imbalance Hard Physical effort
A_MU4	- Good phase in speed: possibility of luffing more than Luca and the other riders - Gain more ground to windward than Luca in the gust	Good speed Improving position in the fleet
A_MU5	- Entering a lull, slowing down, loss of power in the sail - Loss of flight height	Decreasing wind Decreasing speed Decreasing sail power Decreasing flight height
A_MU6	- Sudden feeling of stalling of the sail (sail backwinded, loss of power) - Loss of windward ground compared to the other riders after the bear away (10 meters bear away)	Perturbating sail movements Decreasing sail power Worsening position in the fleet
A_MU7	- Significant physical effort to constrain the flight (sensation of having difficulties) - Lower back pain - Loss of windward ground (“falls” on Luca) - Very light wind	Hard physical effort Worsening position in the fleet Light wind
A_MU8	- The wind is coming back (puff) - Sensations of balance of the forces transmission association with a “well placed” equipment	Increasing wind variation Transmission balance
A_MU9	- Contrasted sensations of moments when the sail naturally “sucks” forward, and allows to be “well settled,” and moments when there is not this tendency	Transmission balance

**Table 7:** Categorization of Lucas’ typical representamen.

MU	Representamen	Typical content of the Representamen
L_MU1	- Gathering of all riders, placed on the same line perpendicular to the wind - Is placed leeward of the fleet	Maintaining position in the fleet
L_MU2	- Riders flying - All placed on a line perpendicular to the wind	Maintaining position in the fleet
L_MU3	- All riders flying - All riders get going	Maintaining position in the fleet
L_MU4	- Slower than the other riders - Light wind	Worsening position in the fleet Light wind
L_MU5	- Well balanced sail, stable, “solid” - Board acceleration	Transmission balance Increasing speed
L_MU6	- “Good sensations” of balanced transmission and downforce on the board - *“Good phase in sensation”*	Transmission balance
L_MU7	- Good sensations of balanced transmission in the puff - “Neutral” sail - Acceleration of the board (hissing of the foil) - Equipment” *climbs in a rather natural way”*	Transmission balance Increasing speed Transmission balance
L_MU8	- Increase of the lift (“*the wind lifts a lot*”) - Increasing lateral gap with respect to the other riders	Rotating wind Worsening position in the fleet
L_MU9	- Decrease of the wind speed (“lighter”) and the traction of the sail - After adjustment of the settings: - The transmission (downforce) is no longer centered in the harness lines - The leech gets “heavier” - Less “natural” transmission, unbalanced: stronger traction with the rear arm, push on the front arm	Decreasing wind Transmission imbalance
L_MU10	- Wind speed remains low - Loss of speed and flight height (limit to touch down) - Greater stress on the rear leg (calf) (less balanced transmission, les “comfortable”)	Light wind Decreasing speed Transmission imbalance
L_MU11	- Puff: entering the puff	Increasing wind
L_MU12	- Wind a little more to the right than at the start of the speed test (header) - An “improved photo” (position in relation to the other riders) compared to the previous evaluation	Rotating wind Improving position in the fleet

The analysis reveals eight typical contents of the riders’ representamen during the speed-test: flight height, physical effort, position in the fleet, sail movements, sail power, speed variations, transmission balance, and wind variations. These typical contents could be categorized by referring to dichotomous perspectives that traditionally distinguishes the attentional foci to be internal versus external, broad versus narrow, associative versus dissociative, and proximal versus distal (e.g., Wulf, 2013; Bernier et al., 2016). But it is noteworthy that most representamen aggregate several typical contents (belonging in some cases to opposite dichotomous categories) into complex perceptual experiences, that make sense as totalities for the athletes. For example, in his course-of-experience, Adam applied force on his back foot to maintain the flight, with a feeling of being in a bad phase (A_MU7). This meaningful unit of experience is connected to a representamen that includes hard physical effort, decreasing sail power and worsening position in the fleet. That is, at this moment Adam is simultaneously paying attention to his body sensations (e.g., the physical effort to apply force on his back foot to constraint the flight), the environmental conditions (at the moment he is in a lull), and the consequences of the situation (i.e., worsening position in the fleet). Thus, most representamen show a distribution of the attentional foci simultaneously on various and heterogeneous elements of the performance environment, implying a multisensory integration that makes sense for the rider. These observations are in line with results of previous research highlighting the multidimensionality of foci of attention (Bernier et al., 2016), emphasizing the limits of dichotomous approaches of attentional focus in sport. Our results are also consistent with those of Pluijms et al. (2016), which revealed that sailing performance is not correlated with external nor internal foci of attention. These authors suggest that expert sailors’ activity differs from less skilled sailors’ activity by integrating multisensory information to guide their actions, as this integration is deemed essential for the control of motor action in three particular domains: aviation, sports, and driving (Gray, 2008).

Furthermore, the analysis reveals that the typical contents of the riders’ representamen have a valence that can be described by adverbs and adjectives such as: increasing or decreasing (speed, wind, flight height); improving or worsening (position in the fleet); hard or easy (physical effort); balanced or imbalanced (transmission). On the one hand, this reflects a sensitivity to the elements of the situation that provide information for assessing the moment-to-moment performance. On the other hand, this reflects active expectations about situations judged as favorable or unfavorable for performance. Athletes’ ongoing self-assessment of performance during movement execution has been shown by previous studies in acrobatic sports (e.g., Hauw et al., 2003, 2008; Nyberg, 2014), or in rowing (e.g., Millar et al., 2017). Moreover, in an experimental setting, Ioannucci et al. (2021) showed an association between motor learning and the capacity of the subjects to judge their movements fluidity. The results of our analysis suggest that IQfoil riders assess their performance by being sensitive to variations with respect to a set of personal experiential references that form “norms of performance” for them. For example, the moment that Adam qualifies as “the worst” is in fact the combination of hard physical effort, worsening position in the fleet and light wind (A_MU7). That is, self-assessment of performance in IQfoil is made pre-reflectively in relation to situated subjective norms (Rietveld, 2008)^6^ rather than in reference to objective criteria of performance measurement which are impossible to take into account in real situations in sailing (Terrien et al., 2023).

### 4.3 A micro-scale phenomenological description of continuous improvement

Studying the activity of freeskiers, Nyberg (2014) showed that experts have developed a capability to discern and modify their velocity during their movements (“tricks”). For Nyberg (2014), skiers’ previous experiences are integrated into a “subsidiary knowing” that shapes and creates embodied frames of references from which it is possible to discern their velocity. In the course-of-experience framework, a local analysis of the interpretant provides information on continuous knowledge validation and emergence in sport situations (e.g., Sève et al., 2002; Bourbousson et al., 2011). In the present study, we analyzed the interpretants by identifying their involvement in the rider’s “frame of reference,” shaping the ongoing action. We found nine categories, referring to four domains of knowledge: (a) Training Procedures; (b) Technical Skills; (c) Tactical Skills; (d) Performance Assessment. (Tables 8, 9).

**Table 8:** Adam’s dynamic of knowledge validation, invalidation or emergence during the speed test.

MU	Interpretant	Categories	Domain of knowledge
A_MU1	- Procedure-type of starting a speed test	Reinforcement of a training organizational routine	Training procedures
A_MU2	- Technical adaptation-type: tighten outhaul entering a gust	Reinforcement of a usual technical skill	Technical skills
A_MU3	- It is not necessary to reduce the power of the sail in these conditions to reach optimum speed, as long as it is possible to “counter” the overpower	Emergence of context of validity of a technical skill	Technical skills
A_MU4	- The advantage taken over the other riders is due to good technical use of the gust (allowing him to luff and gain more ground windward), and not to a wind shift (“alignment”)	Reinforcement of a typical principle of interpretation of a tactical situation	Tactical skills
A_MU5	- Technical adaptation-type: releasing outhaul restores power in the sail	Reinforcement of a usual technical skill	Technical skills
A_MU6	- Releasing too much outhaul can cause the sail to stall - Reaction-type to emergency situation: bear away sharply to re-attach the airflow to the sail	Emergence of context of validity of a technical skill Reinforcement of a usual technical skill	Technical skills
A_MU7	- Meaningful “worst moment” experienced in the search for performance in an upwind leg	Emergence of a situated norm of performance assessment	Performance assessment
A_MU8	- The outhaul was too loose in the lull - Technical adaptation-type: when the wind comes back everything gets back into place with the new setting	Emergence of context of validity of a technical skill Reinforcement of a usual technical skill	Technical skills
A_MU9	- In the good phases, the sail “aspirates” forward naturally and allows to be settled easily; it is possible to accompany the sail with the arms when it settles naturally and to accentuate this tendency; when the sail does not “aspirate” naturally, it would be impossible to constrain it to do so.	Reinforcement of a situated norm of performance assessment	Performance assessment

**Table 9:** Luca’s dynamic of knowledge validation, invalidation or emergence during the speed test.

MU	Interpretant	Categories	Domain of knowledge
L_MU1	- Procedure-type of starting a speed test	Reinforcement of a training organizational routine	Training procedures
L_MU2	- Procedure-type of starting a speed test	Reinforcement of a training organizational routine	Training procedures
L_MU3	- Procedure-type of starting a speed test	Reinforcement of a training organizational routine	Training procedures
L_MU4	- Slower speed that the other riders - Wind “lighter” for him than for the others	Emergence of a situated norm of performance assessment	Performance assessment
L_MU5	- Optimal sail settings indicators-types	Reinforcement of a situated norm of performance assessment	Performance assessment
L_MU6	- Experience-type of stable and balanced transmission	Reinforcement of a situated norm of performance assessment	Performance assessment
L_MU7	- A balanced transmission allows the equipment to adapt “in a natural way” to wind variations (without having to compensate, possibility of “letting the equipment do its thing”)	Reinforcement of a situated norm of performance assessment	Performance assessment
L_MU8	- Wind lift is unfavorable in a leeward position of the fleet	Reinforcement of a typical principle of interpretation of a tactical situation	Tactical skills
L_MU9	- An optimal adjustment of the outhaul is one that makes it possible to obtain a sail that pulls more, while maintaining a balance of transmission (without needing to compensate by pulling with the rear arm)	Emergence of a new technical skill	Technical skills
L_MU10	- Scenario-type of having to “force the flight” in critical flight conditions	Reinforcement of context of validity of a technical skill	Technical skills
L_MU11	- Scenario-type: “getting things going again in a puff”	Reinforcement of context of validity of a technical skill	Technical skills
L_MU12	- Leeward rider is advantaged when wind heading during a speed test	Reinforcement of context of validity of a technical skill	Tactical skills

The interpretants referring to Training Procedures belong to a single category: “reinforcement of a training organizational routine.”

The interpretants referring to Technical Skills fell into four categories: “Reinforcement of a usual technical skill,” “Reinforcement of context of validity of a technical skill”; “Emergence of context of validity of a technical skill,” and “Emergence of a new technical skill.”

The interpretants referring to Tactical Skills belong to a single category: “Reinforcement of a typical principle of interpretation of a tactical situation.”

The interpretants referring to Performance Assessment fell into two categories: “Reinforcement of a situated norm of performance assessment,” and “Emergence of a situated norm of performance assessment.”

The number and the plurality of categories of interpretants referring to technical skills and performance assessment require special attention. Indeed, this analysis reveals that knowledge relating to the technical skills is not simply “applied” nor reinforced. It is constantly being transformed and under construction. The categories “Emergence of context of validity of a technical skill,” and “Emergence of a new technical skill,” are particularly interesting in this respect. For example, a context of validity of a technical skill emerged in Adam’s course-of-experience with the interpretant associated to A_MU3. At this moment, Adam’s interpretant is that it is not necessary to reduce the power of the sail in these conditions to reach optimum speed, as long as it is possible to counter the overpower. That is, at this moment, Adam became aware of the context of validity in which to make use of technical skills he already had (i.e., wind conditions, sea conditions, equipment tuning and settings, as well as Adam’s personal conditions, offering to him the possibility to counter the sail’s power without modifying the settings). On other occasions, new skilled solutions (i.e., solutions never tried before) were implemented. This is the case in Luca’s interpretant associated with L_MU9. At this moment, Luca learnt to finely trim the outhaul while maintaining a balance in the transmission of force, feeling that the good adjustment is when he does not need to compensate with the arms (i.e., the usual solution in this kind of situation). Overall, the categories referring to technical skills reflect the great flexibility and the very contextualized and contingent nature of the technical skills mobilized by the experts in their ongoing activity during training sessions. Each situation thus gives athletes the opportunity either to consolidate technical skills and their context (more or less broad) of implementation, or to limit this context of implementation to a smaller class of situation, or even to build new technical skills more appropriate to new or unfamiliar circumstances.

A similar analysis could be developed with regard to the categories of interpretants concerning performance assessment. These reveal the alternation during the riders’ activity between the conjunctural emergence of situated norms of performance assessment and the reinforcement of these norms. For example, in Adam’s course-of-experience, the interpretant related to A_MU7 is an emerging situated norm of performance assessment. Indeed, at this moment a new reference of the “worst moment”of the upwind leg emerged in Adam’s course-of-experience. This reference creates a new basis to assess the quality of the following experiences, or to reassess previous good or bad phases experienced. These categories reinforce the idea that, like the other categories of knowledge, the personal experiential reference that constitutes “norms of performance” for riders is constantly being refined and constructed in the ongoing riders’ performance optimization.

The least represented domains of knowledge are training procedures and tactical skills. Even though it is important to note that this quantitative consideration is based on a data sample limited in size, this observation is easily explained if we consider two characteristics of the situation analyzed (speed-test upwind). Firstly, this training task is a very simple one in terms of its procedure. Secondly, one of the conditions required to guarantee the relevance of a speed-test in sailing is to neutralize the tactical stakes of the situation, by making it compulsory to sail on a specific tack and point of sail. This could explain why the only tactical knowledge reinforced by the riders is to interpret circumstantial evolutions of position in the fleet during the speed-test.

In the introductory chapter of the book “The phenomenology of continuous improvement,” Toner et al. (2021) give the examples of some world-class elite athletes in various sports who continually seek to refine or change their motor skills or techniques over their careers. For example, they report that despite having won eight medals at the Beijing Olympics, Michael Phelps (the most successful Olympian swimmer of all time) decided to change his freestyle technique in a bid to increase his sprinting speed. For these authors, the process of continuous improvement is the phenomenon whereby certain elite sport performers are able to continuously improve their skills through deliberate practice, even after they have become experts. However, these authors mainly considered this process in broad temporal scales (e.g., several years, one Olympiad).

We put forward the hypothesis that the dynamic of knowledge reinforcement, transformation or emergence that we have described, which accompanies the optimization of their performance in the athletes’ courses-of-experience, is a phenomenological description of “continuous improvement” at a micro-scale level (the ongoing activity). In other words, in the present case study, we illustrated how continuous improvement phenomena can be studied at a micro-scale level, as a micro-genesis of continuous skill acquisition and adaptation over time, using the course-of-experience framework. This approach complements longitudinal approaches to understand how the development and discovery of new solutions at a micro-temporal scale translate into long-term improvement.

## 5 Conclusion: characterizing the experience of being absorbed in an activity of performance optimization

This article aimed to illustrate the fruitfulness of the theoretical and methodological approach of the course-of-experience to describe and characterize the pre-reflective self-consciousness that is involved in the activity of ongoing performance optimization and improvement in elite athletes. The empirical analysis highlighted (a) the variety of meaningful activities in which the athletes are engaged when seeking to optimize their performance, allowing them to adapt flexibly to their unpredictable and dynamic performance environment; (b) the multidimensionality of the athletes’ attentional foci, and the dynamics of their moment-to-moment subjective performance self-assessment; and (c) the dynamics of knowledge reinforcement, transformation or emergence accompanying their activity. These dimensions seem to be fundamental to consider if one wishes to understand the activity of optimizing performance “in-the-doing” from the viewpoint of the athlete. Indeed, it reflects what it means for an athlete to be “absorbed” in the ongoing activity of performance optimization (Colombetti, 2011). In line with Colombetti (2011), we consider that this absorption implies neither the inconspicuousness nor the fully implicit character of body perceptual experiences and motor-skill regulations. On the contrary, it implies a rich and dense experience in which body awareness and situation awareness alternate at a pre-reflective level that characterizes the lived experience of performance optimization. From a practical standpoint, this position is consistent with training interventions that utilize mindfulness-based methods aiming at developing self-awareness and cognitive flexibility (e.g., Gardner and Moore, 2017; Carraça et al., 2018). As the debate continues on the relation between consciousness and skilled action (e.g., Foultier, 2023), we claim that further exploration of these pre-reflective dimensions of consciousness is a fruitful direction for future research and applied implications in the domain of human performance in cognitive sciences. In this perspective, the course-of-experience framework offers researchers theoretical and methodological tools to conduct empirical investigations according to enactive and phenomenological assumptions.

## Data availability statement

The raw data supporting the conclusions of this article will be made available by the authors, without undue reservation.

## Ethics statement

The studies involving humans were approved by Comité d’éthique pour la recherche non interventionnelle, Nantes Université. The studies were conducted in accordance with the local legislation and institutional requirements. The participants provided their written informed consent to participate in this study.

## Author contributions

ET: Conceptualization, Data curation, Formal analysis, Investigation, Methodology, Writing – original draft, Writing – review & editing. BH: Conceptualization, Data curation, Formal analysis, Investigation, Methodology, Writing – original draft, Writing – review & editing. PI: Data curation, Funding acquisition, Writing – original draft, Writing – review & editing. JS: Conceptualization, Data curation, Formal analysis, Funding acquisition, Investigation, Methodology, Supervision, Validation, Writing – original draft, Writing – review & editing.

## Footnotes

^1^It should be noted that some authors use the notions of reflective and pre-reflective “consciousness” or “self-consciousness” (Legrand, 2007; Poizat et al., 2023a), while other authors use the terms “awareness” or “self-awareness” (Colombetti, 2011; Toner et al., 2016). Moreover, some authors refer to these phenomena in terms of “bodily self-awareness” (or self-consciousness), to highlight the embodied and situated nature of the human experience.

^2^For a detailed presentation of this enactivist approach to practice and cognition in practice, and its phenomenological and semiotic inspirations, see Poizat et al. (2023a).

^3^IQfoil was the Olympic sailboard class at the time of the study.

^4^It is important to emphasize that the notion of knowledge is linked here to a non-representationist (or non-mentalist) conception of knowledge and human cognition. In reference to the theoretical background of the course-of-experience framework, the notion of knowledge must be conceived as “elements of generality” grounded in embodied experiences. This notion of embodied knowledge emphasizes the actor’s construction of regularities or generalizations based on the discovery of typicality in their relationships with the world from past and present experiences. From this perspective, the Referential consists of the actor’s activation of usual knowledge (or types), i.e., the embodied habits inherited from past experiences; and the Interpretant consists of the process of validation or development of usual knowledge, or of enacting new types. The Interpretant “allows for the continual transformation of the Referential, which reveals learning over the course-of-experience” (Poizat et al., 2023a,b, p. 116).

^5^The neologism representamen comes from Peirce’s sign-thought theory. It can be compared to the notion of affordance, from the ecological theory of perception (e.g., Gibson, 1979), but with the consideration that there is no “direct input” of “information.” The selection by the actor of the elements of the situation which are relevant to them, as well as the meaning for the actor of what they perceive, results at each moment from their involvement in the situation, their structure of anticipation and from their referential.”

^6^For Rietveld, “The notion of normativity […] is a very basic one, namely distinguishing adequate from inadequate, correct from incorrect, or better from worse in the context of a particular situation.” (Rietveld, 2008, 973).
