# Supplementary material for: Documenting and analyzing pre-reflective self-consciousness underlying ongoing performance optimization in elite athletes: the theoretical and methodological approach of the course-of-experience framework
Source: Front Psychol. 2024 Jun 25;15:1382892. doi: 10.3389/fpsyg.2024.1382892 (PMC11231638; doi:10.3389/fpsyg.2024.1382892)
Supplement: Supplementary file 1 [file Table_1.DOCX]

**Appendix A**

**Protocols to analyze to riders’ courses of experience**

Note: The original work documents have been translated from French to English by the authors for the purpose of the article. Original French versions are available upon request.

**Table A1**

*Protocol to analyze Adam’s course of experience*

| **Time**  **hh:mm:ss** | **In situ spontaneous comments and events** | **Corresponding excerpts of self-confrontation interview** |
| --- | --- | --- |
| 14 :52 :05 | Preparation of the upwind port tack speed test. Adam is positioned leeward of the fleet and windward of Lucas  *((repeated glances at the other riders, windward))*  ***“ Is it good ? “***  Start of the upwind port tack speed test | **(…)**  **Video play**  RES: So the start of the upwind [leg], here we are still on port [tack]  ADAM: That's the second [speed test] yeah?  RES: that's the second speed test, yeah, you have Luca leeward and Tim windward |
| 14 :52 :22 | ***“ Let’s go, we’re about to hit a gust, I think I will be overpowered so I apply a little more out-haul”***    *(( pulls the rope along the wishbone to apply out-haul))* |  |
| 14 :52 :48 | ***"It's going well, I'm a bit overpowered, there's a bit of back hand, but it's okay, I manage to counter it, it still accelerates well"***  *((glances at the other riders))* | **Video paused :** ***"It's going well, I'm a bit overpowered, there's a bit of back hand…”***  RES: You say “a bit of back hand but it’s okay I manage to counter it…”  ADAM: Well, I was at the limit in fact of…, I remember well I was really at the limit of the power that I manage to hold to keep up performance I think. Meaning, if I flattened more, I don’t think it would have been… [I would have been] maybe a bit more comfortable but I wouldn’t have performed better but... , so here I am really at this limit of… I don't need to flatten, it's physical there, because I have to hold on a bit but I don't need to re-flatten because anyway…, I think anyway I’m at the optimum speed!  RES: and so that's what you, you're looking to get this maximum power limit before having to be …, and what's your threshold, well, at what point you say to yourself okay well there is no need to flatten, that’s to say, you feel [it] then...  ADAM: well, I feel it’s powerful, but I still manage to counter, to counter this power, and to transform it into speed.  RES: ok, but countering this power… how do you feel this power? In the rear hand traction, in...?  ADAM: well in fact, the wishbone yeah you feel that there is power coming in the back hand, a little on the leech of the sail, and that it is starting to open up a little and even to lift a little from the back  RES: ok, do you feel that the boom tends to go up like that…?  ADAM: to open up and raise a little  RES: okay, and that you have to resist that…  ADAM: and I have to counter it yeah  RES: ok, and so in fact you tell yourself that it's time to apply again a bit of out-hall when uh… when it's too [powerful to counter]  ADAM: When it's too much like that yeah exactly…  RES: and when it's too much like that it's... what do you feel...?  ADAM: [when it's too powerful counter] you feel the top of the sail al little backwinded, and there I think [it's the case] a little bit, but it's still bearable, and above all, you feel that with a less powerful sail you could accelerate...  RES: ok and you can feel that…well, you consider that as long as you can keep a lot of power and that you can resist physically, it's good…?  ADAM: yeah  RES: ok, and physically… what is it? Do you get tired the back arm tired ? Earlier you said here I am settled I am balanced, do you feel now that it is not [the case] anymore?  ADAM: Yeah it's a little less balanced, I'm pushing my back hand more than [if I was balanced] that's really where I'm doing the physical effort... is really in that back hand now…  RES: Is this physical effort tiring?  ADAM: yeah it's tiring you can't hold [long], here like that I'm at the limit yeah, on a short leg of the course ok, but if I have to do a raid, if I have to do a 10-minute leg I apply a bit more [outhaul] to be able to hold…  **Video play**  RES: ok, so here we are moving forward, so here finally you are, you are almost a little overpowered in the sail  ADAM: I'm almost overpowered yeah  RES: in the sail  ADAM: in the sail yeah |
| 14 :53 :20 | *((looks behind him in direction of the other riders))*  ***“ it’s going well, they are lining up behind, it looks like I’m doing the spoon. Well here uh…technical uh…  “***  *((Look up at the top of the sail))*  ***“ I have the sail well settled, the foil I have power, the glide isn’t too bad”»*** | **Video paused**  RES: [the alignment] you perceive it as a, I don't know…, as a confirmation of what you were expecting earlier that it was going, it was going to shift left [the wind]?  ADAM: Yeah maybe yeah yeah…yes…I kind of feel like that, and like I had a good phase I think…I didn't say anything but I think [I was in a good phase ]  RES: Then it's, it's rather... it lines up [all the boards] uh..., it [the wind] heads on port there...  ADAM: Yeah uh, no, [it’s] more [like] touching the gust a bit more… no I think it's more in relation to the good phase that I had, where I think in the gust I have maybe a small speed advantage, and there I had the impression that I was able to [luff], that it served me [in windward gain possibilities]…, so just maybe it's just an impression eh…  RES: okay  ADAM: And compared to Luca also I felt like I was coming back a bit, so I felt like I was in a good phase…  RES: to come back…, that is to say?  ADAM: No…I think he was a bit like that at the start of the upwind leg there [mimes with his hands the respective positions of his board and of Luca’s], and there I have the impression of having done that a bit on him [mimics a movement of his board rising upwind in relation to Luca's], less exaggerated but a little...  RES: ok, to have passed a little more windward  ADAM: to have, passed a little more windward, and to go up a little, in speed... |
| 14 :53 :38 | ***“In the lulls I tend to go down a little on the foil… “***  ***« there it’s mellow, we’re going to stop… It’s slowing down a lot“***  *((Looks behind him))*  ***« I’m going to release some outhaul, hop… »***  *((loosen the outhaul))* | **Video play : *“In the lulls I tend to go down a little on the foil… “***  RES: Ok, go ahead, comment… yeah, so there…  ADAM: Well there was the wind transition [decrease of the wind speed] and I think I may have eased [loosen the tension in the sail], I think I straightened up to release the boom at the time of the transition [release outhaul from the boom]  **Replay video "I'm going to release some outhaul…"** |
| 14 :53 :51 | ***Oh damn I almost lost the wind, I have to bear away there, it’s just awful***  *((look behind him))* | **Video paused : *« It’s just awful »***  and…, I remember at that moment, the sail stalled…, yeah the wind detached from the sail, so I had to bear away again to attach everything back into the sail, all the wind…  RES: ok, and when you say uh, the wind has detached from the sail, that is to say, you really feel the stall, it's..., what is it... how do you feel about that? It's in terms of...  ADAM: … of power in the sail, I feel that …  RES: ok… is it really decreasing?  ADAM: yeah it definitely decreases [the power], even if I remember well, there it almost backwinded [reverse incidence of the wind in the sail], well I felt that…, there was a moment when I had nothing left [no power] in the sail, and after that it didn't... it almost pushed from the other side, I had to bear away hard, that's what I say [in the microphone] I had to bear away hard to [recover]  RES: to recover, attach the air flow..., and when you say "it's just awful" is that the phenomenon?  ADAM: well yeah that was hard, well yeah it's, I had the impression of having to fall again ten meters downwind, well to re-attach the wind, well really not to stop !  RES: yeah that's what you say: "I almost lost the wind"  ADAM: yeah  RES: okay  ADAM: It's that the wind almost backwinded [the sail] |
| 14 :54 :15 | Loses ground to Luca  ***“There it’s hard, my lower back hurt, there in front there’s no air, really no air…”***  ***“it’s coming back”*** | **Video play**  RES: and there we hear your breathing again, to breathe does that…  **Video paused: *“There it’s hard, my lower back hurt, there in front there’s no air, really no air…”***  RES: ok when you say your lower back hurts in front…  ADAM: Well that's the feeling you get when it's physical, it pulls in the back here [points to the painful area in the lower back] so I had on this phase… where I “ was falling” on Luca [loss of ground compared to Luca, in a leeward position], but I still wanted to stay close-hauled … There was less wind, much less wind, it was starting to strain here [points to lower back]  RES: yeah… and compared to earlier, the stance, finally you're still a lot on the back foot?  ADAM: There in the lull I'm a lot [on the] back foot yeah, I try to be enough back foot to give lift [to the foil]  RES: yeah, and always with this…, this desire to have…?  ADAM: yeah I [don't] think so, when it's light wind, well, in fact I force to sail like that [gesture to move the sail forward] when I feel good, but there I was too constrained everywhere to be able to do that, I was already just trying to get the board to fly correctly to say to myself "there I'm going to optimize my sail placement", I was already physically fighting too much for this...  RES: ok, so you were trying to constrain the board, stay in the air and get the sail re-attached [to the airflow]…  ADAM: That's it, re-attach, get back on a good system as they say where, finally… where everything is back in place and let’s go again!  RES: OK, and what is this good system? At other times do you get it in this leg?  ADAM: I think that, from memory, this is the worst part of the session… it's now !  RES: okay  ADAM: Oh no, there's another moment when I'm with Tom too when I say it's difficult, these are the two moments that I found difficult in the session, and only one during the downwind (…) but here is the worst  RES: this is the worst leg upwind  ADAM: The worst leg of the… of the year! Anyway, it's not the worst leg, it's the worst moment because at the beginning I had some great phases... now, that moment was the worst moment! |
| 14 :54 :29 | Hit a puff  ***“Here it comes back. I tighten back a little outhaul, I had let go a little too extreme”***  *((applies more outhaul))* | **Video play : *“It’s coming back… Here it comes back”***  ADAM: yeah, you can even see it on the video  RES: we see, what do we see… is it the low flight? what are you referring to?  ADAM: well there, even from the angle…, from the attitude of the equipment  **video break**  ADAM: well, I find that the equipment is “placed” (…) here we can see when the wind comes back, there, everything is back in place, I find that it can be seen not too bad [points to the screen video] |
| 14 :54 :40 | At the end of the speed-test, while continuing to sail, comments aloud on his impressions of the whole leg (personal mini-assessment)  ***"It's not bad today, the thing that I manage to feel a little is that I manage to bring the mast forward there in the right phases"***  ***"try to really that..., the front of the sail pushes, at the same time when I feel that it does that I try to accentuate it a little with my arms so this is not bad"*** | **Video play *“It’s not bad today…”***  RES: ok that's what you were saying earlier...  ADAM: in the god phases  RES: so there you were debriefing  ADAM: A little debriefing of the leg yeah…  **Video paused**  ADAM: So yeah, that was a bit of the debriefing of what I was saying, that is, in the right phases there, in fact the sail, sometimes, the sail as I was saying, it aspirates you in a little forward and in in fact, it allows you to settle in a little better and it aspirates you in… in fact in these moments, when I feel that the material does it a little naturally, I try to accompany it, but on the other hand when it [doesn’t] I don't want to do it I [don't] force him to do it because I think that means that…well, I would have difficulty in forcing him, well physically I [can't]…, but in any case when I feel it does it I try to accentuate it… |
| 14 :55 :05 | *((TACK))* – End of the speed test – gathering around the coach boat. |  |

**Table A2**

*Protocol to analyze Luca’s course of experience*

| **Time hh:m:ss** | **In situ spontaneous comments and events** | **Corresponding excerpts of self-confrontation interview** |
| --- | --- | --- |
| 14 :49 :53 | Gathering of the riders near the coach's boat. The athletes trim their sails and prepare to perform a speed test on port tack. Luca positions himself leeward of the other riders, and on the tie line (perpendicular to the wind) before starting. | **(…)**  **Vide paused on the group of riders before the start of the speed test**  RES: ok so we're going to start the first port tack speed test, so try to put yourself back in the situation, we started with a port tack speed test, I don't know where you were...  LUCA: I was leeward  RES: oh yes you were leeward  RES: ok yeah and besides there was also Adam, you were leeward Adam and you  LUCA: yeah  RES: ok, on this speed test do you remember significant things for you? |
| 14 :51 :48 | Get started with the port tack speed test *((pumping movements of the sail))*  Beginning of the flight. Luca remains on par with the others until the speed test starts *((frequent glances at opponents))* | **Video play**  RES: I let you comment on what you are doing, at this moment, what you are trying to do, what you perceive, what you are doing, what you may be saying to yourself at this moment... |
| 14 :52 :10 | ***“Ok let’s go!”*** (Start of the speed test) |  |
| 14 :52 :17 | ***“Pretty slow so far,*** *((glances towards windward riders))*  ***good puff coming in front…***  ***It’s starting to speed up***  ***I re-tighten the outhaul”****((tighten the outhaul))* | **Video paused : *« Pretty slow so far, good puff coming*… »**  RES: ok, so at this point…  LUCA: well, start of the leg I think the wind is a little light, I see a puff coming in front of us so a little bit windward I'm trying to adapt the setting of my sail that is to tighten quite a lot the outhaul.  RES: yeah, quite a lot…?  LUCA: well, practically flat out, but that is to say above all to have the feeling of having a sail that is fairly neutral, which carries me but which is fairly neutral in terms of stability, that is to say that allows me to open up if, to open up a little in phases where I'm going to try to luff up a little bit, or in any case to gain upwind uh, and that it's really uh, now there's a fairly well-centered downforce in the harness lines, so what I mention at one point and uh well for me it's a downforce which is important, that means that the foil and the equipment develop power and I manage to channel that and to retransmit that uh, a little in the opposite direction of the harness lines.  RES: and so before, before doing this adjustment to re-stabilize your rig, do you anticipate the instability that there could be during the puff or do you already feel something at this moment?  LUCA: no, I don't feel instability at this moment... well that was yeterday’s feeling it's that the puffs they were, it's a bit very patchy on the water so, when you enter the puffs sometimes they are quite unstable so you don't know which way the wind was going to be when entering the puffs so my objective is above all to be very very stable and in some way solid when entering the puff so as not to be destabilized and to be very efficient right away, not to have a sail opening movement or I'll lose a few seconds of acceleration things like that so uh, that's why I try a lot of anticipate uh, on the settings and I don’t wait to be in the puff to make the adjustment because I would have had to open my sail a little.  RES: ok and here during the leg it can happen to you, to adjust several times on a speed test like that, over 2-3 minutes you can tighten loosen your leech several times?  LUCA: yeah that was the case yesterday (previous training) there were still quite a few moments when the wind was lighter so then I loosen a little bit and really just entering the pressure I was trying to anticipate a little bit the set up… |
| 14 :52 :30 | ***“Here we go, I’m on my way !...***  ***Good downforce in the harness lines…***  ***Rather well balanced, I am light on the arms… “*** | **Video play: “good downforce in the harness lines… rather well balanced”**  RES: ok, so wait **(video pause)**, when you say “good downforce in the harness lines”… “light on the arms, balanced…”  LUCA: hm, well that's what I was kind of saying in fact, I really try to make adjustments so that the forces are rather centered so that the force really passes through there and that I don't have to compensate for sail settings or rig positions through the arms by actually being able to get into position, release the sail and just lean against the gear and have the main transmission just through the harness lines. I don't have to tense up or adapt with my arms which would mean that I would necessarily remove the pressure in the harness lines  RES: okay, and when you say "good downforce in the harness lines" that's what you're saying there, that is to say centered, the fact that you don't have any pressure, well you don't have to compensate with the arms etc. and have it centered but is there something else you feel…?  LUCA: well, that is it carries me uh, just by the pelvis and by the support of the harness line in fact, I mean I feel that at the same time I am being lifted but at the same time I am also weighing in on that point and that I am strong there, and not in the arms to compensate, and that allows me to let my sail, I mean, to adjust it in a neutral way, that the effort applies on this point and that it also allows me to put myself in the position of being able to extend my arms and also to be able to put my body windward and to weigh, to put some righting moment in the position...  RES: yeah okay and when you say "*it allows me both to be stable, to feel that it pulls me well on this point and also allows me to weigh down*", there is something that you feel in particular? For example, do you feel the harness in a particular way? Your pelvis…?  LUCA: well yeah the forces are there, but it presses slightly more on the front side of the harness, I feel that I'm a little more globally compressing the front like the foil, the equipment is trying to push me up, well of course I adapt a little, in general it is quite balanced but it is slightly more on the front buttock...  RES: ok, ok and being more forward, do you also feel a difference in pressure on the legs?…  LUCA: no, unlike downwind, I don't saturate the front leg or anything, it's more of a balance where I manage, once the settings are done correctly, you just have to regulate a little more the height of flight, there is a little more general tension in the front leg but it's not over-stressing, it's not tiring, unlike earlier downwind or uh, yesterday I thought to myself at the end of the leg, yeah the thigh was hurting. This is not the case after the speed test...  **Video play** |
| 14 :52 :44 | ***“The wind lifts…”***  ***“ The phase is quite good in sensations…”*** | RES: So when [the wind] lifts, how do you see it, it's just modifying...  LUCA: well, it's sensations precisely during the puff when the trim of the sail is good I am able to open my sail a little bit and in fact my board immediately climbs in the wind, well, after that I don't know I haven't checked on the camera on the left if I'm doing a comparison, but hey, I feel that as it accelerates, we're gaining into the wind of the uh..  **Video break: “*The phase is quite good in sensations…*”**  RES: yeah at this point you say “the phase is pretty good in sensations…”  LUCA: well that's more because actually I find that I'm pretty well balanced in my settings there's nothing that bothers me and in fact I'm able to open the sail a little bit and that all. that retransmits in the board, I mean the equipment luffs and, and climbs in a rather natural way I am not obliged to compensate…, to force on one arm or the other, in fact my sail is rather neutral, “hop” I can open it a little bit because I feel that there is more pressure and the wind is rather to the left so it [lifts]…  RES: yeah, that's kind of what you mentioned earlier, the balance you keep in the harness... And when you say "the phase is good in sensations", is it there's something about, I don't know, about the quality of your glide, about, I don't know, something..., in terms of...  LUCA: no, well, that's... we hear it a little from the sound, we hear the foil a little more, so it's the equipment that accelerates... For me, it's always well balanced, that is to say that there I place my sail, the puff hits, the equipment it luffs, I mean I, I have no contrary force nor I feel that I have to balance myself in a way uh, a little particular to make good use of the conditions. |
| 14 :53 :20 | ***“The wind lifts a lot”  ((look at the others windward))***  ***“I try to stay in a high mode”*** | **Video play.**  RES: ok, so we're going to let it play, you can comment if there are things that are significant for you...  LUCA: well, I felt that I also had a little less wind than the others so it was climbing a little more and there was a quite a bit of lateral gap that was created with the others  RES: when you say it was climbing it was climbing above you  LUCA: yeah  RES: we do not have the impression on the video there [that it climbs]…  LUCA: well on the tracks [shows the GPS tracks on the screen] we can still see that the gap is [increasing]…  **Video break.**  LUCA: so there precisely to limit uh, the possible loss or the gap to the others, well I try to stay in high mode precisely for..., if there are puffs that fall, well so I too can take advantage of it and so there isn't really a shear, and I [don’t] completely change of puff or mode so uh…that’s it, the logic is to try to stay high enough and uh, well after I anticipate a little but uh, the "photo" will improve precisely for me at the end of the speed test so..., to do that I think I limit the bleeding a little, especially if it is going to improve it will improve again to my advantage rather than theirs [the other riders].  RES: if it heads a little yeah…  **Video play.** |
| 14 :53 :37 | ***“Lighter [the wind]”***  ***“I loosen outhaul…*** *((loosen the leech))* ***it’s very mellow [the wind]!”*** | LUCA: so there really the puff stops a little bit… so there I put a little bit of power again  RES: OK. And there the fact of releasing the outhaul a little, what does it change? Do you feel that it changes, I don't know, well is it to restore power? You said, does that change things in terms of I don't know, a little more support behind, a little more support, well I don't know how you...  LUCA: yeah well... (sigh), it's something I need to improve but in general, when you release the outhaul you feel that you put a little volume back in the top of the sail, and immediately you feel that it pulls a little more upwards from the rig  RES: Really? Okay  LUCA: yeah, after uh, it's really light, you must not let go too much because as we are still going fast you also feel that you immediately also increase the drag on the sail and that for once you can quickly get to…  RES: do you feel? what do you feel?  **Video paused.**  LUCA: well, the leech which is getting a little heavier, the need to have to use the rear arm more to sheet in and that the adjustment is a little less natural…  RES: less natural means you have to…  LUCA: yeah to compensate with the arms yeah, the downforce is no longer essentially centered on the ends of the harness, it's... I compensate by having to pull on my rear arm to precisely sheet int the sail a little more...  RES: um…, are you trying to focus on something, on the sheeting…? What are you focusing on?  LUCA: well it's essentially the back hand which... after that it's a little more a movement of... [shows gesture] it pushes the front arm slightly and you try to pull more with the back arm  RES: okay, it's more the feeling you have in your arms, you don't have… you don't look at your leech…  LUCA: no, so I'm trying to adjust the trim to find the outhaul tension that keeps carrying it upwards, but I'm actually able to keep the sail neutral, and not compensate. The moment when I compensate I think is the moment when I have a little... when the adjustment is less precise, and perhaps worse, well that's what I try to concentrate on when I regulate at the moment… afterwards it may not be good but uh, for me it's the thing that I try to look after in terms of sensations  **Video play.**  RES: ok, we are moving forward, so there... |
| 14 :53 :51 | ***“Fairly low flight, the wind is light”***  *((look at riders windward))*  ***“I step back a little with my back foot to try to put a little more pressure on the foil”*** | RES: "fairly low flight, the wind is light", so there...  LUCA: there on this phase however I am a little obliged to, there we see my board it is really at the limit of touching at a moment I am obliged a little to boost the foil with my back foot so that solicits me a little more the, well the calf the back leg, precisely because I may be moving my foot back a little bit, I must say, maybe not on this speed test there, but another time when I will try to more constrain to precisely try to give back a little upforce and fly a little higher to feel that I'm not at the limit of touching, really almost, not to stop but to slow down  RES: so that's what you said a bit earlier... And here how... it's a feeling that you would describe how, it's a little, it's a little less pleasant, it's a little more, I don't know?  **Video break.**  LUCA: yeah, well there, regarding the comment I made on the back foot, there we really see that the board sometimes goes down quite low, well we feel that it's becoming almost limit to fly, but in any case it's a a little more…, it's a little harder to really go upwind so…  RES: you have to force the…  LUCA: yeah a little bit and you can see precisely the moment I find where I comment there on the back foot I find that the board takes a little height for a few lengths, so it's not bad but uh, there I'm mostly in the observation of the wind, regarding the wind, uh, well wait for a pressure which will come back in order to be a little more comfortable and uh, and the same in the observation, that is telling me when the rise will hit [and] how am I going to be effective quickly, to adapt the setting and that it accelerates immediately. |
| 14 :54 :23 | *“Puff to come”*  *“ I begin to touch [the wind]”*  *“a bit more”*  *“that’s it”*  *((look at the windward riders))* | RES: comment if there something that…  **Video play**  RES: ok, then, you have already commented the entry in the puff…  LUCA: Nope |
| 14 :54 :49 | *((look at the windward riders))* ***“wind a little more to the right than at the beginning of the speed test, it improved the picture for me”***  *((repeated glances windward))* | RES: yeah so it heads a little  LUCA: In fact there may have been a header related to the lull but uh… |
|  | *((TACK))* *–* End of the speed test – Gathering around the coach boat. | RES: yeah. So on that leg...  **Video break.**  LUCA: ((spontaneous report of the speed test)) well uh, good…, well, rather good feelings when the pressure is up, but it's always difficult when you're leeward to really analyze your capacity, well really your pure speed we can say uh, compared to the others because you have no direct vision, on the opponents so…, I rather tried, although when I said my adjustment was perhaps not very good at the beginning of the session for once it may have helped me to stay high enough precisely not to change of system or puff from the others who were windward and uh, in any case regarding the sensations when there was really strong wind strong during the leg, I was pretty, I felt pretty good.  (…) |
