# Supplementary material for: Documenting and analyzing pre-reflective self-consciousness underlying ongoing performance optimization in elite athletes: the theoretical and methodological approach of the course-of-experience framework
Source: Front Psychol. 2024 Jun 25;15:1382892. doi: 10.3389/fpsyg.2024.1382892 (PMC11231638; doi:10.3389/fpsyg.2024.1382892)
Supplement: Supplementary file 2 [file Table_2.DOCX]

**APPENDIX B**

**Riders’ hexadic signs identification**

Note: The original work documents have been translated from French to English by the authors for the purpose of the article. Original French versions are available upon request.

**Table B1**

*Adam’s hexadic signs*

| **Time (hh:m:ss)** | **Meaningful units (MU)** | **Involvement** | **Expectations** | **Referential** | **Representamen** | **Interpretant** |
| --- | --- | --- | --- | --- | --- | --- |
| 14:52 :05 | A_MU1: Gets his board flying upwind, while observing the other riders | - To take the start of the speed test  - To control the flight before the start of the speed test  - To stay on the same line as the others for the start | - All riders flying on a same line, ready to start the speed test (“is it good?”) | - Typical procedure: A ST requires that all riders start equally, on the same line perpendicular to the wind | - positioning on the same perpendicular line to the wind  In a leeward position of the fleet, and windward of Luca | - procedure-type of starting a speed test |
| 14 :52 :22 | A_MU2: Tighten his outhaul | - To reduce the “power” of his sail before hitting the gust | - is going to enter in gust  - the gust will “overpower” his sail | - to apply more outhaul prevents the sail to overpower | - incoming gust | - technical adaptation-type: tighten outhaul entering a gust |
| 14 :52 :48 | A_MU3: Accelerates in the gust while countering the sail and telling himself that he has reached the limit of the sail power allowing him to remain efficient | - To obtain and maintain an optimal VMG  - To use the gust to accelerate  - To evaluate the need (or not) to adjust the setting of the sail. | - Acceleration in the gust  - Need (or not) to flatten again the sail (applying more outhaul)  - too much effort (or not) to “counter” the sail power | - To accelerate the sail must not be too powerful  - Typical indicators of sail power: back hand traction, leech opening  - On a short course, I can withstand the effort required | - optimal speed  - High traction in the back hand (powerful sail)  - The sail slightly backwinded but remains “bearable” (allows to withstand and to maintain an optimal speed)  - feeling of imbalance between the tractions in the two hands  - Sensations of effort and physical fatigue to “counter” the overpower | - it is not necessary to reduce the power of the sail in these conditions to reach optimum speed, as long as it is possible to “counter” the overpower |
| 14 :53 :20 | A_MU4: Visually checks the other riders and then his sail, telling himself that he is in a “good phase” of speed | - To assess his trajectory in relation to the other riders  - To visually check the sensation of having a “sail well settled” | - Maintaining a good speed in the gust  - Variation of his placement and speed in relation to the other riders during the gust | - Variations in wind strength and/or direction can benefit more or less to the rider depending on their placement | - Good phase in speed: possibility of luffing more than Luca and the other riders  - Gain more ground to windward than Luca in the gust | - the advantage taken over the other riders is due to good technical use of the gust (allowing him to luff and gain more ground windward), and not to a wind shift (“alignment”) |
| 14 :53 :38 | A_MU5: Releases outhaul tension in the lull, while controlling his placement in relation to the other riders | To adapt his sail settings to the decrease of the wind to maintain speed | - marked slowdown in the lull  - loss of flight height | - the foil loses power in the lull (tendency to “go down a little”)  - releasing the outhaul tension restores power in the sail in the lulls (when the wind dies down) | - entering a lull, slowing down, loss of power in the sail  - loss of flight height | - technical adaptation-type: releasing outhaul restores power in the sail |
| 14 :53 :51 | A_MU6: Suddenly bears away surprised by the stalling of the sail, and controls the loss of ground to the other riders | - To quickly react to the stalling of the sail to make it re-attach the wind and restore power  - To prevent being stopped while the sail is stalled | - loss of “wind” (the sail is stalling)  - loss of speed and flight height | - the sail “stalls” when the incidence of the wind is too low  - the sail can be backwinded when the angle of attack is tool low, causing a sudden drop of power in the sail  - bearing away allows to open the sail an re-attach the airflow to it | - sudden feeling of stalling of the sail (sail backwinded, loss of power)  . loss of windward ground compared to the other riders after the bear away (10 meters bear away) | - releasing too much outhaul can cause the sail to stall  - reaction-type to emergency situation: bear away sharply to re-attach the airflow to the sail |
| 14 :54 :15 | A_MU7: Forces on the back foot to maintain the flight, with a feeling of being in difficulty, in a “bad phase” | - to maintain the flight in the lull by applying maximal constrain on the board  - to make the sail reattached to the airflow  - to keep heading windward despite the loss of speed | - loss of flight and loss of windward ground in the lull  - a “good system” of operation | - In the lull the priority is to keep flying and not to optimize the sail positioning  - typical experiences related to a “good operating system” and “degraded” operation | - significant physical effort to constrain the flight (sensation of having difficulties)  - lower back pain  - loss of windward ground (“falls” on Luca)  - very light wind | - meaningful “worst moment” experienced in the search for performance in an upwind leg |
| 14 :54 :29 | A_MU8: Modifies sail settings as he enters the puff, thinking he had previously released outhaul “too extremely” | - To adapt the sail settings to the increase of the wind (puff)  - To find a “good operating system” | - restoration of power in the equipment  - a “good system” of operation | - applying more outhaul redistributes the power in the sail in the puff (when the wind increases) | - the wind is coming back (puff)  - sensations of balance of the forces transmission association with a “well placed” equipment | - the outhaul was too loose in the lull  - technical adaptation-type: when the wind comes back everything gets back into place with the new setting |
| 14 :54 :40 | A_MU9: At the end of the speed test, makes a “report” aloud of his impressions | To verbalize the main lessons learned from the speed test | - new knowledge to optimize his operation | - typical experiences related to a “good operating system" and to a “degraded” operation  - typical experience related to the “natural tendency” of the sail to “aspirate” (or not) forward. | - contrasted sensations of moments when the sail naturally “aspirates” forward, and allows to be “well settled”, and moments when there is not this tendency | - in the good phases, the sail “aspirates” forward naturally and allows to be settled easily; it is possible to accompany the sail with the arms when it settles naturally and to accentuate this tendency; when the sail does not “aspirate” naturally, it would be impossible to constrain it to do so. |
| 14 :55 :05 | A_MU10: Tacks (end of the speed test) |  |  |  |  |  |

**Table B2**

*Luca’s hexadic signs*

| **Time (hh:m:ss)** | **Meaningful units (MU)** | **Involvement** | **Expectations** | **Referential** | **Representamen** | **Interpretant** |
| --- | --- | --- | --- | --- | --- | --- |
| 14 :49 :53 | L_MU1: Position himself leeward of the other riders, on a same line | - To prepare for the start of the speed test  - to be on par with the other riders at the start | - all riders on a same line | - Typical procedure : a speed test start requires that all riders are on a sane line, perpendicular to the wind | - gathering of all riders, placed on the same line perpendicular to the wind  . is placed leeward of the fleet | - procedure-type of starting a speed test |
| 14 :51 :48 | L_MU2: Pump and start flying while observing the other riders | - To position himself and control the flight before the start of the speed test  - To stay on par with the other riders | - all riders flying on a same line, ready to start the speed test | - starting a speed test in IQF requires all riders flying | - riders flying  - all placed on a line perpendicular to the wind | - procedure-type of starting a speed test |
| 14 :52 :10 | L_MU3: Begins the speed test  « *Ok let’s go*» | - To obtain and maintain an optimal VMG  - To take the start of the speed test | - Optimal VMG | - The speed test begins when all riders are flying on a same line | - all riders flying  - all riders get going | - procedure-type of starting a speed test |
| 14 :52 :17 | L_MU4: Assess his speed in relation to the others (look at the others “*Pretty slow so far*”) | - to estimates his performance (speed, windward ground gain) compared to the windward riders | - position of advantage over the other riders | - the windward ground gained is estimated in relation to a perpendicular to the wind  - all riders may not benefit from the same wind during a speed test | - slower than the other riders  - light wind | - slower speed that the other riders  - wind “lighter” for him than for the others |
|  | L_MU5: Negotiates the entry into a puff by adapting his outhaul setting | - To make good use of the puff to optimize his VMG  - to adjust the sail settings to higher wind conditions | - incoming puff  Neutral sail, settled, allowing to be well balanced and to accelerate when entering the puff | - important to adapt the settings to the wind variations  - a well set sail = neutral and centered in the harness lines, allowing easy sheet in/sheet out, and good transmission of the thrust to the board | - well balanced sail, stable, “solid”  - board acceleration | - optimal sail settings indicators-types |
| 14 :52 :30 | L_MU6: Optimize his operation in the new conditions | - To maintain an optimal operation, balanced and stable  . to control flight height | - balance of forces transmission requiring no physically expensive compensation *“just by the pelvis and harness lines”*  - postural stability  - stable flight height | - typical experiences of balanced and stable transmission of forces: downforce in the harness lines, sensation of transmission (pelvis, pressure on the front of the harness…), lightness in the arms (no twitching) | - “good sensations” of balanced transmission and downforce on the board  *-“good phase in sensation”* | - experience-type of stable and balanced transmission |
| 14 :52 :44 | L_MU7: Negotiates a lift (evolution of its position compared to the others): opens his sail slightly and lets the board luff to follow the wind variation | - To follow to rotation of the wind maintaining optimal, balanced and stable operation | - balance of transmission  - following the wind shift  - maintaining of an optimal speed | - When transmission is balanced, it is easy to open the sail and follow the wind shift | - good sensations of balanced transmission in the puff  - “neutral” sail  - acceleration of the board (hissing of the foil)  - equipment *”climbs in a rather natural way”* | - a balanced transmission allows the equipment to adapt “in a natural way” to wind variations (without having to compensate, possibility of “letting the equipment do its thing”) |
| 14 :53 :20 | L_MU8: Says to himself that the wind lifts a lot and that he has “less wind than the others”, and opts for a “high mode” | - to evaluate his position at time t compared to the other riders  - to stay in “high mode” (close to the wind) to maintain the lateral gap with other riders  - to take advantage of the same puff as the other riders | - reduction of the lateral gap with the other riders despite the lift | - a wind lift is unfavorable in a position leeward of the fleet (it increases the lateral gap)  - in a speed test, the riders may not benefit all from the same wind conditions, when there is a significant lateral difference between the, (“shear” phenomenon) | - increase of the lift (“*the wind lifts a lot*”)  - increasing lateral gap with respect to the other riders | - wind lift is unfavorable in a leeward position of the fleet |
| 14 :53 :37 | L_MU9: Release outhaul tension (perceiving a decrease of the wind speed) | - To set the sail to regain a bit more power | - increased sail traction  - maintenance of a balance of transmission  (“neutral” sail)  - need (or not) to compensate for transmission imbalance (front/rear arm traction) | - releasing outhaul puts more volume in the top of the sail and increases the force in the sail  - releasing outhaul can cause a transmission imbalance that needs to be compensated (pull of the rear arm, push of the front arm) | - decrease of the wind speed (“lighter”) and the traction of the sail  After adjustment of the settings:  - the transmission (downforce) is no longer centered in the harness lines  - the leech gets “heavier”  - less “natural” transmission, unbalanced: stronger traction with the rear arm, push on the front arm | - an optimal adjustment of the outhaul is one that makes it possible to obtain a sail that pulls more, while maintaining a balance of transmission (without needing to compensate by pulling with the rear arm) |
| 14 :53 :51 | L_MU10: Steps back with the back foot (perceiving a loss of flight height), observing the gusts to come | - to get the foil going again to regain flight height  - to anticipate the puffs to reaccelerate immediately | - regain flight height  - possibility of touching down (or not): flight limit, slowing down  - increase of wind speed (puff) allowing to maintain the flight more “comfortably” | - putting more pressure on the back foot allow to increase the flight height  - typical scenario of having to “force the flight” in critical conditions to fly | - wind speed remains low  - loss of speed and flight height (limit to touch down)  - greater stress on the rear leg (calf) (less balanced transmission, les “comfortable”) | - scenario-type of having to “force the flight” in critical flight conditions |
| 14 :54 :23 | L_MU11: Negotiates the entry in a puff | - To make good use of the puff to accelerate | - acceleration  - to regain flight height | - Entering a puff allows to accelerate and fly more easily | - puff: entering the puff | - scenario-type: “getting things going again in a puff” |
| 14 :54 :49 | L_MU12: Observes his position in relation to the other riders, and (perceiving a header) thinks that his position has improved | - To draw conclusion about the speed test | - understand what factors have participated to his performance during that speed test | - The positions between riders can fluctuate depending on the gusts  - a header is favorable to the rider in a leeward position of the fleet in a speed test | - wind a little more to the right than at the start of the speed test (header)  - an “improved photo” (position in relation to the other riders) compared to the previous evaluation | - leeward rider is advantaged when wind heading during a speed test |
